# HIF-1α/MMP-9 Axis Is Required in the Early Phases of Skeletal Myoblast Differentiation under Normoxia Condition In Vitro

**DOI:** 10.3390/cells12242851

**Published:** 2023-12-16

**Authors:** Flaminia Chellini, Alessia Tani, Martina Parigi, Francesco Palmieri, Rachele Garella, Sandra Zecchi-Orlandini, Roberta Squecco, Chiara Sassoli

**Affiliations:** 1 Department of Experimental and Clinical Medicine, Section of Anatomy and Histology, Imaging Platform, University of Florence, 50134 Florence, Italy; flaminia.chellini@unifi.it (F.C.); alessia.tani@unifi.it (A.T.); martina.parigi@unifi.it (M.P.); sandra.zecchi@unifi.it (S.Z.-O.); 2 Department of Experimental and Clinical Medicine, Section of Physiological Sciences, University of Florence, 50134 Florence, Italy; francesco.palmieri@unifi.it (F.P.); rachele.garella@unifi.it (R.G.)

**Keywords:** electrophysiology, HIF, immunofluorescence, MMP-9, myogenesis, myoblasts, normoxia, regeneration, satellite cells, skeletal muscle

## Abstract

Hypoxia-inducible factor (HIF)-1α represents an oxygen-sensitive subunit of HIF transcriptional factor, which is usually degraded in normoxia and stabilized in hypoxia to regulate several target gene expressions. Nevertheless, in the skeletal muscle satellite stem cells (SCs), an oxygen level-independent regulation of HIF-1α has been observed. Although HIF-1α has been highlighted as a SC function regulator, its spatio-temporal expression and role during myogenic progression remain controversial. Herein, using biomolecular, biochemical, morphological and electrophysiological analyses, we analyzed HIF-1α expression, localization and role in differentiating murine C2C12 myoblasts and SCs under normoxia. In addition, we evaluated the role of matrix metalloproteinase (MMP)-9 as an HIF-1α effector, considering that MMP-9 is involved in myogenesis and is an HIF-1α target in different cell types. HIF-1α expression increased after 24/48 h of differentiating culture and tended to decline after 72 h/5 days. Committed and proliferating mononuclear myoblasts exhibited nuclear HIF-1α expression. Differently, the more differentiated elongated and parallel-aligned cells, which are likely ready to fuse with each other, show a mainly cytoplasmic localization of the factor. Multinucleated myotubes displayed both nuclear and cytoplasmic HIF-1α expression. The MMP-9 and MyoD (myogenic activation marker) expression synchronized with that of HIF-1α, increasing after 24 h of differentiation. By means of silencing HIF-1α and MMP-9 by short-interfering RNA and MMP-9 pharmacological inhibition, this study unraveled MMP-9’s role as an HIF-1α downstream effector and the fact that the HIF-1α/MMP-9 axis is essential in morpho-functional cell myogenic commitment.

## 1. Introduction

Postnatal mammalian skeletal muscle growth, maintenance and regeneration after damage are mostly dependent on the activity of satellite cells (SCs), the resident stem cell population [1,2]. In healthy muscle, these mononucleated cells are located in a specialized niche at the periphery of adult striated multinucleated myofibers (hence the name) between the sarcolemma and the basal lamina ensheathing each myofiber in a dormant state. In response to different biochemical and mechanical stimuli emanated by the surrounding growing or injured microenvironment, SCs become activated, exit the quiescent state and start to proliferate and differentiate, essentially recapitulating embryonal myogenesis steps [3,4,5]. SCs express the paired box transcription factor Pax7, regarded as the canonical SC-specific marker necessary for their survival and functionality, and myogenic regulatory factors (MRFs) such as MyoD, myogenin, Myf5 and MRF4. In particular, when quiescent, SCs express Pax7 and Myf5, the transcription factor typical of myogenic lineage, that is usually detected in about 90% of quiescent SCs. Once activated, SCs divide by mitosis giving rise to a progeny of proliferating adult myoblasts that simultaneously express Pax7, Myf5 and the myogenic determinant factor 1 (MyoD), the master regulator of SC proliferation/activation. Afterwards, most of these cells no longer express Pax7, but maintain MyoD and proceed into their myogenic program acquiring myogenin and MRF4/Myf6 expression to differentiate into skeletal myocytes. These, in turn, fuse with each other to form myotubes and finally new myofibers. At the same time, a small population of cells maintains Pax7, down-regulates MyoD and self-renews, not executing the myogenic program. In this way, they reconstitute a pool of quiescent ‘stem cells’ to be recruited on the basis of future demand [6]. Similarly to other stem cells, SCs undergo an energy metabolic reprogramming during differentiation, consisting of a shift in the use of metabolic substrate. In particular, proliferating myoblasts produce energy mainly through glycolysis, which is different to myotubes which exhibit a major aerobic capacity [7,8,9]. Several papers highlighted the hypoxia-inducible factor (HIF)-1α as a regulator of myogenesis and SCs/myoblasts’ homeostasis in vitro and in vivo [10,11,12,13,14,15,16,17,18,19,20,21,22]. It is a dimeric, basic helix–loop–helix PER-ARNT-SIM transcriptional factor composed of a HIF-1β subunit that is constitutively expressed and a HIF-1α (or its paralogs HIF-2α and HIF-3α) subunit that is oxygen sensitive. HIF-1α’s biological activity depends on post-translational modifications that are responsible for the α-subunit stability and activity. Usually, in normoxia, post translational modifications of HIF-1α (HIF-1α is hydroxylated by the active prolyl-hydroxylases -PHDs-) lead to subunit degradation via the ubiquitin–proteasome pathway. By contrast, in hypoxia, PHDs are inhibited, hydroxylation cannot take place and HIF-1α becomes stable so that it can translocate to the cell nucleus. Here, the HIF-1α subunit assembles a transcriptional complex with HIF-1β which is able to interact with different coactivators and to modulate the expression of several target genes implied in many cellular processes comprising the regulation of glycolytic metabolism in skeletal muscle [15,23,24,25]. However, in skeletal muscle tissue and myoblasts/SCs, an oxygen level-independent regulation of the expression and activation of HIF-1α has been reported, thus suggesting a role of such a protein in skeletal muscle physiological functions and homeostasis [10,18,21,22,26,27,28,29,30,31]. Nevertheless, some controversy remains about HIF-1α’s spatio-temporal expression and on its exact role in myoblasts during myogenic differentiation. In addition, knowledge of the molecular targets of this factor needs to be expanded [17,19,32,33].

This study was planned to better clarify the role of HIF-1α in myoblasts undergoing myogenic differentiation under normoxia conditions and to evaluate MMP-9 as a downstream effector of HIF-1α. In particular, we investigated MMP-9 on the basis of the following considerations: (i) MMP-9 was shown to be a target of HIF-1α in different cell types [34,35,36,37]; (ii) a previous study from our research group demonstrated MMP-9 expression in differentiating myoblasts [38]; (iii) although the involvement of MMP-9 in skeletal myogenesis has been described, its regulation and role need to be fully clarified [39,40,41]. Here we demonstrate that: (i) HIF-1α expression has the same time course as MMP-9, peaking during the early phase of differentiation (24 h) concomitantly to that of MyoD; (ii) MMP-9 is an effector of HIF-1α and (iii) HIF-1α/MMP-9 axis is necessary for the morpho-functional myogenic activation/commitment of myoblasts.

## 2. Materials and Methods

### 2.1. Cell Culture and Treatments

Skeletal myoblasts from the murine C2C12 cell line (American Type Culture Collection-ATCC; Manassas, VA, USA), were cultured in proliferation medium (PM) consisting of DMEM supplemented with 10% FBS (Sigma-Aldrich, Milan, Italy) and 1% penicillin/streptomycin (Sigma-Aldrich) under 5% CO_2_ and 95% air in a humidified incubator at 37 °C. To promote myogenic progression, the cells were cultured in PM until 80% confluence was reached. Then they were moved into myogenic differentiation medium (DM) consisting of DMEM supplemented with 2% horse serum (HS; Sigma-Aldrich) for different lengths of time (24, 48, 72 h and 5 days). In some experiments C2C12 cells were cultured in PM or in DM for 24 h in the presence of an MMP-9 inhibitor, SB-3CT (10 μM; Sigma-Aldrich), as previously reported [38].

Murine primary SCs were isolated from single myofibers of *Extensor Digitorum Longus* (EDL) of male Swiss adult mice (25–30 g) according to previously published protocol [5] (n = 3 animals; 24 muscle fibers for every muscle). Briefly, EDL muscles were digested in DMEM with 0.2% collagenase type I (Sigma-Aldrich) added. To isolate single living myofibers from each other, a gentle mechanical trituration was made with a Pasteur pipette in serum-free culture medium. They were then individually transferred to a 24-well plate treated with Matrigel (BD Biosciences, San Jose, CA, USA). The myofibers were maintained for 48 h in the specific SC growth medium containing DMEM plus 20% FBS, 10% HS, 0.5% chicken embryo extract (BioIVT, West Sussex, UK) and 1% penicillin/streptomycin, to let the SCs sprout. Thereafter the myofibers were taken out and the sprouted cells were cultured until 80% confluence was reached (3–5 days) before being detached with a solution containing 0.05% trypsin–0.03% ethylenediaminetetraacetic acid (Sigma-Aldrich). Then, they were seeded (again) in a culture dish containing fresh PM for 20 min. After 20 min, the cells that were not adherent (SCs) were gathered together and seeded on gelatin-coated glass coverslips. They were maintained in SC growth medium for 24 and 48 h until they were subjected to confocal immunofluorescence microscopy analysis.

### 2.2. Silencing of HIF-1a and MMP-9 by Short Interfering RNA

C2C12 cells seeded either on sterile glass coverslips placed at the bottom of a well of a 6-well culture plate or directly on the well of a 6-well culture plate were cultured in PM till reaching 60–80% confluence before being transfected with a pool of 3 short target-specific 20–25 nucleotides from interfering RNA duplexes (siRNA; 20 nM, Santa Cruz Biotechnology, Santa Cruz, CA, USA) designed to knock-down HIF-1α (sc-35562; Sense: 5′CACCAUGAUAUGUUUACUATT3′, Antisense: 5′UAGUAAACAUAUCAUGGUGTT3′, Sense: 5′CCAGUUGAAUCUUCAGAUATT3′, Antisense: 5′UAUCUGAAGAUUCAACUGGTT3′, Sense: 5′CCACUUUGAAUCAAAGAAATT3′, Antisense: 5′UUUCUUUGAUUCAAAGUGGTT3′) or MMP-9 gene expression (sc-29401; Sense: 5′GCUUCCCUCUGAAUAAAGATT3′, Antisense: 5′UCUUUAUUCAGAGGGAAGCTT3′, Sense: 5′CUUCCAGUACCAAGACAAATT3′, Antisense: 5′UUUGUCUUGGUACUGGAAGTT3′; Sense: 5′CAGCUACUUUAGUCAAUCATT3′, Antisense: 5′UGAUUGACUAAAGUAGCUGTT3′). The cells transfected with a non-specific scrambled (SCR)-siRNA (20 nM, Santa Cruz Biotechnology, sc-37007) were used as control. To reach a higher efficiency of inhibition of HIF-1α expression, two consecutive transfections with siRNA were performed 24 h apart as indicated by Ono et al. [21]. In particular, the cells were incubated with HIF-1α-siRNA duplexes or SCR-siRNA for 24 h, and the following day, the transfection was performed again for an additional 24 h, according to the manufacturer’s instructions.

MMP-9 siRNA transfections were carried similarly to previously published protocols [42]. After transfection—PM (T0)—, the medium was changed and the cells were cultured in DM for 24 h or 48 h before being processed for Western blotting, immunofluorescence and electrophysiological analyses.

### 2.3. Morphological Analyses

#### 2.3.1. Phase Contrast Microscopy

Myotube formation after 5 days of C2C12 cell culture in DM was evaluated under an inverted phase contrast microscopy (20×; Nikon Diaphot 300).

#### 2.3.2. Confocal Laser Scanning Microscopy

Cells plated on glass coverslips were fixed with 0.5% PFA at room temperature for 10 min and then processed for indirect immunostaining as in a previously reported protocol [43]. The employed primary antibodies (4 °C; overnight) are indicated in Table 1.

To reveal the immunoreactions, the specific secondary antibodies anti-rabbit, anti-mouse Alexa Fluor 488- or anti-mouse 568-conjugated IgG (1:200; Molecular Probes, Eugene, OR, USA) were used at RT for 1 h.

To reveal F-actin filaments, the cells were incubated with Alexa Fluor 488-labeled phalloidin (1:400 for 20 min at room temperature; Molecular Probes). Negative controls were carried out by replacing primary antibodies with non-immune serum; the cross-reactivity of the secondary antibodies was assessed by omitting the primary antibodies. The glass coverslips with the immunolabeled cells were mounted with an antifade mounting medium (Biomeda Gel mount; Electron Microscopy Sciences, Foster City, CA, USA). Observations were performed under a confocal Leica TCS SP5 microscope equipped with a HeNe/Ar laser source for fluorescence measurements and differential interference contrast (DIC) optics, (Leica Microsystems, Mannheim, Germany) by using a Leica Plan Apo 63×/1.43 NA oil immersion objective. A series of optical sections (1024 × 1024 pixels each; pixel size 204.3 nm) 0.4 μm in thickness were taken throughout the depth of the cells at intervals of 0.4 μm and were projected onto a single ‘extended focus’ image. Quantitative analysis of Ki67- and MyoD- positive nuclei was performed by counting the positive cells in at least 5 random 200 × 200 μm^2^ square microscopic fields (63× objective) in each cell preparation and the results were reported as the percentage of positive nuclei on the total cell nuclei. Densitometric analyses of the mean fluorescent signal intensity for each specific marker were performed on digitized images using ImageJ 1.49v software (https://imagej.nih.gov/ij/ (accessed on 7 March 2022)) in 10 regions of interest (ROI) of 100 μm^2^ or in 20 ROI of 0.25 μm^2^ for each confocal stack (at least 10). The experiments were all performed in triplicate.

### 2.4. Total RNA Extraction and Semi Quantitative Reverse Transcription (RT)-PCR

Total RNA was extracted from C2C12 cells by using TRIzol Reagent (Invitrogen, Life Technologies, Grand Island, NY, USA), according to the manufacturer’s instructions. Reverse transcription and amplification of 1 µg of total RNA were performed in a thermal cycler (Perkin Elmer, Monza, Italy) using qScript XLT One-Step RT-PCR Kit (Quantabio, Beverly, MA, USA). After a starting cycle at 55 °C for 30 min and 94 °C for 2 min for pre-denaturation, the samples were subjected to 40 cycles of PCR, alternating between 94 °C for 15 s, 55 °C for 30 s and 72 °C for 1 min; the final extension step was carried out with a cycle at 72 °C for 5 min. The following mouse gene-specific primers were used: HIF-1α (NM_001313919.1), forward 5′-TCA GCA TAC AGT GGC ACT CA-3′ and reverse 5′-AAG GGA GCC ATC ATG TTC CA-3′ (transcript length 213 bp); MMP-9 (NM_013599.4), forward 5′-CTG GCG TGT GAG TTT CCA AA-3′ and reverse 5′-CTA GCA CCT TTC CCT CGG AT-3′ (transcript length 201 bp); β-actin (NM_007393.3), forward 5′-ACT GGG ACG ACA TGG AGA AG-3′ and reverse 5′-ACC AGA GGC ATA CAG GGA CA-3′ (transcript length 206 bp). β-actin mRNA was used as the internal control. Samples with no template (only water) were carried out in each run. The PCR products were separated on 1.8% agarose gel electrophoresis stained with ethidium bromide and detected with a UV transilluminator. Densitometric analysis of the bands was performed by using ImageJ 1.49v software (https://imagej.nih.gov/ij/ (accessed on 7 March 2022)) and normalized respect to β-actin.

### 2.5. Western Blotting

Total proteins were extracted from C2C12 cells grown in the different experimental conditions and quantified as previously reported [42]. Forty micrograms of total proteins were electrophoresed on NuPAGE^®^ 4–12% Bis-Tris Gel (Invitrogen; 200 V, 40 min) and blotted onto polyvinylidene difluoride (PVDF) membranes (Invitrogen; 30 V, 1 h). The membranes were incubated on a rotary shaker first for 30 min at room temperature with the Blocking Solution included in the Western Breeze^®^ Chromogenic Western blot Immunodetection Kit (Invitrogen) and then overnight at 4 °C with the antibodies reported in Table 1. Protein immunodetection was performed according to the Western Breeze^®^Chromogenic Immunodetection protocol (Invitrogen). Densitometric analysis of the bands was performed using ImageJ 1.49v software (https://imagej.nih.gov/ij/ (accessed on 7 March 2022)) and the values were normalized to α-tubulin for each result, assuming α-tubulin as control invariant protein.

### 2.6. Electrophysiological Recordings

C2C12 cells cultured on glass coverslips placed in standard plastic culture dishes (35 mm × 10 mm, Corning Incorporated, Corning, NY, USA) in PM or DM and silenced or not for HIF-1α expression or in the presence or absence of SB-3CT (10 μM), were subjected to electrophysiological analysis as previously reported [44,45]. Bioelectrical features of the cells were investigated using the whole cell patch clamp technique at 22 °C.

The patch electrodes were produced from borosilicate glass capillaries (GC150-15; Clark, Electromedical Instruments, Reading, UK) by a two-step vertical puller (Narishige, Tokyo, Japan) and then filled with the following filling pipette solution (mM): 130 KCl, 10 NaH_2_PO_4_, 0.2 CaCl_2_, 1 EGTA, 5 MgATP and 10 HEPES (pH 7.2 with KOH). The electrode resistance was around 1.5–2 MΩ. The coverslip with the cells was positioned in the experimental chamber on the stage of an inverted microscope (Nikon Eclipse TE200, Amstelveen, NL, USA) filled with the following physiological solution (mM): 150 NaCl, 5 KCl, 2.5 CaCl_2_, 1 MgCl_2_, 10 D-glucose and 10 HEPES (pH 7.4 with NaOH). The experimental apparatus for the electrophysiological records comprised an Axopatch 200 B amplifier, A/D-D/A interfaces Digidata 1200; Pclamp 6 software (Version 6.0, Axon Instruments, Foster City, CA, USA) as described in earlier paper [46]. Data analysis was made by Clampfit 9 software (Version 9.0, Axon Instruments, Foster City, CA, USA). The voltage-clamp mode of the Axopatch amplifier was set to analyze the membrane passive properties: starting from a holding potential (HP) of −70 mV two step voltage pulses of ±10 mV was applied. We calculated the cell linear capacitance (Cm) of the patched cell from the area underneath the capacitive current trace. Since the membrane-specific capacitance is =1 μF/cm^2^ this parameter can be considered indicative of the cell surface area. We also calculated the membrane conductance (Gm) from the steady-state membrane current (Im), as detailed in earlier studies [46], to obtain the specific membrane conductance using the ratio Gm/Cm. This parameter can be considered as an index of the cell permeability. Still in voltage-clamp mode, we carried out the ion currents analysis. Ion currents were evoked by a suitable pulse protocol of stimulation consisting of 1-s step voltage pulses applied from a HP = −80 mV and ranging from −80 to 50 mV, in 10 mV increments. The capacitive and leak currents were eliminated on-line by the P4 procedure. For a correct comparison of the current values recorded from cells of dissimilar dimensions we achieved the current normalization by dividing the amplitude for Cm, so that the resulting I/Cm value (expressed in pA/pF) indicates the current density.

To better provide evidence for the eventual occurrence of Ca^2+^ currents, without different overlapping ion fluxes, we filled the bath recording chamber with a Na+- and K+- free high-TEA external solution (mM): 10 CaCl_2_, 145 tetraethylammonium bromide, 10 HEPES and the patch pipette with a suitable internal solution (mM): 150 CsBr, 5 MgCl_2_, 10 Ethylene-bis(oxyethylenenitrilo) tetraacetic acid (EGTA), 10 (4-(2-hydroxyethyl)-1-piperazineethanesulfonic acid) (HEPES) (pH = 7.2). All of the chemicals were acquired from Sigma-Aldrich.

### 2.7. Statistical Analysis

Data are mean ± SD of at least three independent experiments performed in triplicate. Statistical analysis was performed by one-way ANOVA with post-hoc Tukey HSD test calculator for comparing multiple treatments (https://astatsa.com/OneWay_Anova_with_TukeyHSD/ (accessed on 7 March 2022)) and *p* < 0.05 was considered statistically significant. For the multiple comparisons of electrophysiological data, we used One way ANOVA with Bonferroni’s post hoc test.

## 3. Results

### 3.1. HIF-1α and MMP-9 Expression during C2C12 Myoblast Differentiation under Normoxia

**Figure 1:**
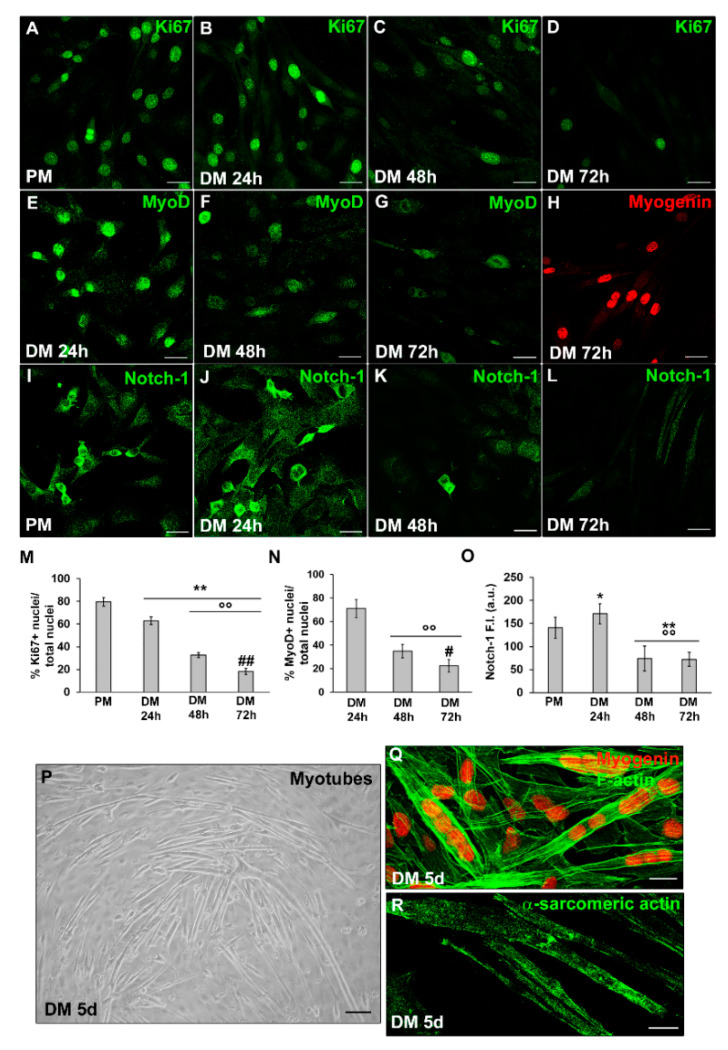
Morphological analysis of C2C12 myoblast differentiation under normoxia. C2C12 cells were cultured in the proliferation medium (PM) for 24 h or in the myogenic differentiation medium (DM) for different time (24, 48, 72 h and 5 d). (**A**–**L**) Representative confocal immunofluorescence images of C2C12 cells immunostained with antibodies against (**A**–**D**) the nuclear protein Ki67 (green), (**E**–**G**) MyoD (green), (**H**) Myogenin (red) and (**I**–**L**) Notch-1 (green). The antibody anti-Notch-1 that we used recognizes both Notch-1 receptor and the Notch intracellular domain (ICD) released from the plasmamembrane after a cleavage in response to Notch activation. (**M**,**N**) Quantitative analyses of Ki67 and MyoD positive nuclei expressed as the percentage of the total nuclei, respectively. (**O**) Densitometric analysis of Notch-1 mean fluorescence signal intensity (F.I.) in the indicated experimental condition, performed on digitized images in 10 regions of interest (ROI) of 100 μm^2^ for each confocal stack (10). a.u.: arbitrary units. (**P**) Representative phase contrast light microscopic image of myotube formation after 5 d culture in DM. (**Q**,**R**) Representative confocal immunofluorescence images of C2C12 cells grown in DM for 5 d immunostained with antibodies against myogenin (red) and sarcomeric actin (green), respectively. In (**Q**) the cells were counterstained with-phalloidin to visualize F-actin filaments (green). Scale bar: in (**A**–**L**,**Q**,**R**), 25 µm; in (**P**), 200 µm. Data are mean ± SD of at least three independent experiments conducted in triplicates. Significance of difference: * *p* < 0.05, ** *p* < 0.01 vs. PM; °° *p* < 0.01 vs. DM 24 h; # *p* < 0.05, ## *p* < 0.01 vs. DM 48 h (One-way ANOVA with post-hoc Tukey HSD).

The myogenic differentiation model of C2C12 cells was constructed and verified through extensive morphological and biochemical analyses (Figure 1).

Accordingly, C2C12 cells were cultured in PM for 24 h and then shifted to DM for different lengths of time (24, 48, 72 h and 5 days) to observe the differentiation, always maintaining physiological oxygen levels. We observed that C2C12 cells after 24 h of culture in differentiation medium (DM, DMEM + 2% HS) exhibited nuclear expression of the proliferation marker Ki67 (Figure 1A,B,M) and of the myogenic commitment marker MyoD (Figure 1E,N). In particular, the number of Ki67^+^ cells appeared to be reduced compared to cells cultured in PM (Figure 1A). Moreover, we observed that these cells showed an increased expression of Notch-1, a key determinant of SC activation, proliferation and self-renewal [47,48], in the cytoplasm and nucleus as compared with the cells in PM (Figure 1I,J,O). The expression of such markers declined with the increase in time (Figure 1A–G,I–O). After 72 h of culture, the cells displayed nuclear expression of myogenin (Figure 1H), the myogenic regulatory factor in terminal differentiation. After 5 days they fuse, resulting in elongated multinucleated myotubes with nuclei positive for myogenin and expressing α-sarcomeric actin (Figure 1P–R), which is strongly indicative of cell myogenic differentiation.

**Figure 2:**
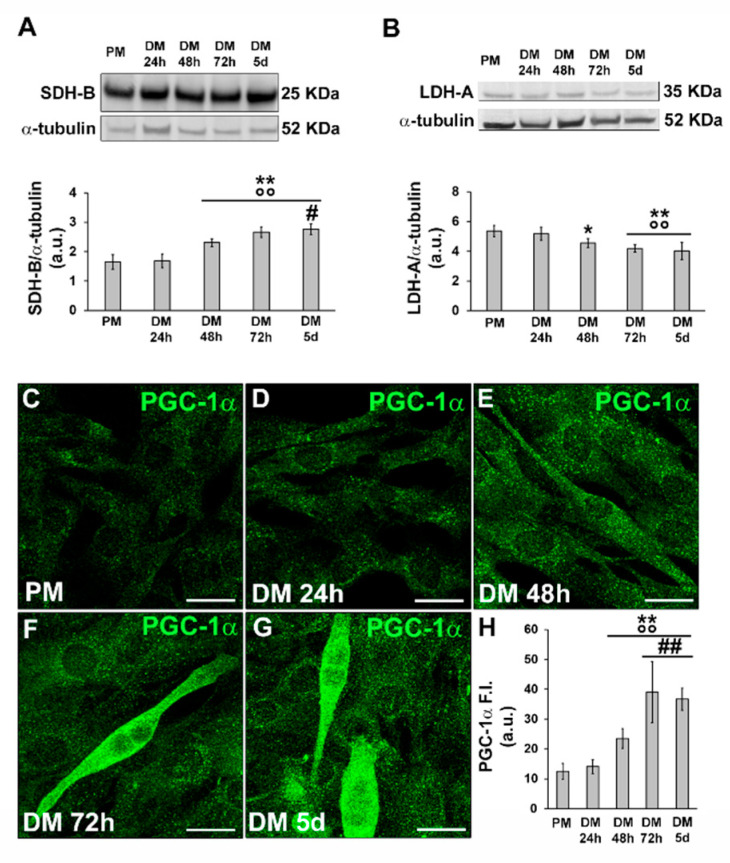
Evaluation of the SDH-B, LDH-A and PGC-1α during C2C12 myoblast differentiation. The cells were cultured in the proliferation medium (PM) for 24 h or in the myogenic differentiation medium (DM) for different lengths of time (24, 48, 72 h and 5 d). (**A**,**B**) Western blot analysis of SDH-B, LDH-A: representative blot and bar charts showing the densitometric analysis of the bands normalized to α-tubulin. a.u.: arbitrary units. (**C**–**G**) Representative confocal fluorescence images of the cells immunostained with antibodies against PGC-1α (green). Scale bar: 25 µm. (**H**) Bar charts showing the densitometric analysis of the PGC-1α mean fluorescent signal intensity (F.I.) performed on digitized images in 10 regions of interest (ROI) of 100 μm^2^ for each confocal stack (10). a.u.: arbitrary units. Data shown are mean ± SD and represent the results of at least three independent experiments performed in triplicate. Significance of difference: * *p* < 0.05, ** *p* < 0.01 vs. PM; °° *p* < 0.01 vs. DM 24 h; # *p* < 0.05, ## *p* < 0.01 vs. DM 48 h (One-way ANOVA with post-hoc Tukey HSD).

Western blot analyses of SDH-B and LDH-A suggested that the cells underwent a metabolic shift during differentiation, from anaerobic glycolysis to the oxidative pathway, confirming previously published data [7,8] and contributing to validating our cell myogenic differentiation model. Indeed, we observed a decrease in the expression of LDH-A over time (Figure 2B) concomitant to an increase in SDH-B expression (Figure 2A).

In line with these observations, the cells also exhibited an increase in the expression of PGC-1α, which is recognized as a key regulator of mitochondrial biogenesis (Figure 2C–H), which reached the maximum expression level in the myotubes (Figure 2F,G).

**Figure 3:**
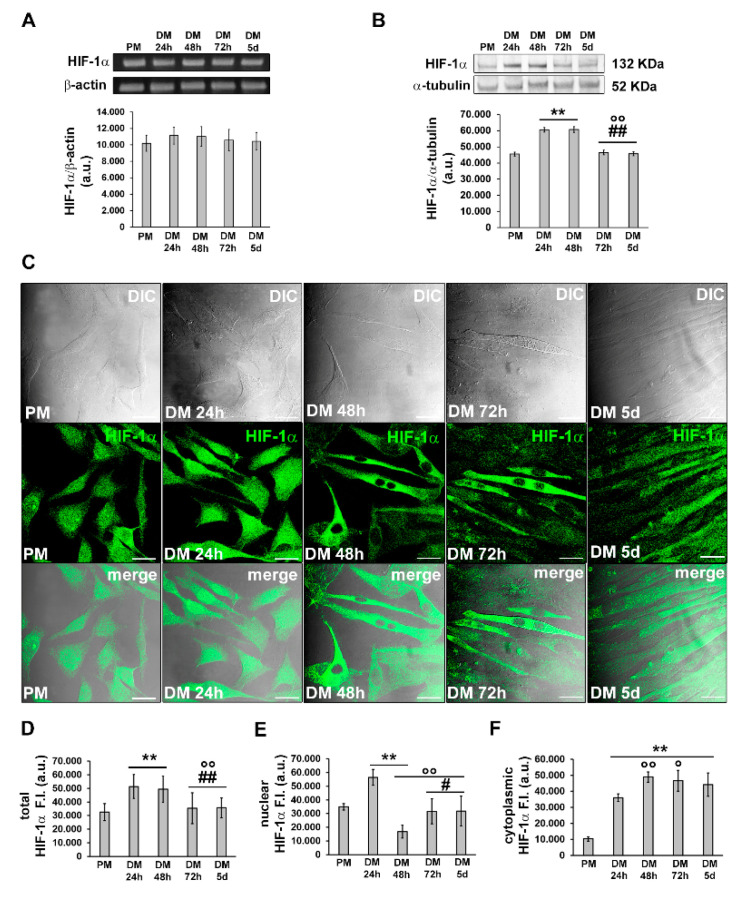
Evaluation of HIF-1*α* expression and cellular localization during C2C12 myoblast differentiation. The cells were cultured in the proliferation medium (PM) for 24 h or in the myogenic differentiation medium (DM) for different times (24, 48, 72 h and 5 d). (**A**) RT-PCR analysis. Representative agarose gel and bar charts showing the densitometric analysis of the bands normalized to β-actin. a.u.: arbitrary units. (**B**) Western blot analysis. Representative blot and bar charts showing the densitometric analysis of the bands normalized to α-tubulin. a.u.: arbitrary units. Data are mean ± SD and represent the results of at least three independent experiments performed in triplicate. (**C**) Confocal microscopy. Upper line: representative differential interference contrast (DIC); middle line: representative confocal immunofluorescence images of cells immunostained with antibodies against HIF-1α (green); lower line: representative superimposed confocal fluorescence and DIC images (acquired simultaneously). Note the nuclear localization of HIF-1α in the early differentiating cells. Scale bar: 25 µm. (**D**–**F**) Bar chart showing the densitometric analysis of (**D**) total HIF-1*α* fluorescent signal intensity (F.I.) performed on digitized images in 10 regions of interest (ROI) of 100 μm^2^ for each confocal stack (10), and (**E**) nuclear and (**F**) cytoplasmic HIF-1*α* F.I. performed on digitized images in 20 ROI of 0.25 μm^2^ for each confocal stack (10). a.u.: arbitrary units. Significance of difference: ** *p* < 0.01 vs. PM; ° *p* < 0.05, °° *p* < 0.01 vs. DM 24 h; # *p* < 0.05, ## *p* < 0.01 vs. DM 48 h (One-way ANOVA with post-hoc Tukey HSD).

Subsequently, we investigated the expression and subcellular localization of HIF-1α during C2C12 myoblast differentiation by RT-PCR, Western blotting and confocal immunofluorescence analyses (Figure 3).

HIF-1α mRNA expression appeared to be constant in C2C12 cells in both the growth and myogenic differentiating phases (Figure 3A), in agreement with previous observations [19,21,28,29]. The protein expression was detected at each time point of differentiation with an increase after 24 h and 48 h of culture in myogenic DM. We observed a tendency towards decline after 72 h and 5 days (Figure 3B). Confocal immunofluorescence analysis revealed that HIF-1α was mainly expressed in the nuclei of proliferating and mononuclear myoblasts committed to the differentiation process (24 h). After 48 h, when the cells became more elongated and more parallel aligned, likely becoming ready to fuse with each other, HIF-1α appeared to be mainly localized in the cytoplasm; in the latest phases of differentiation, myotubes displayed both a cytoplasmic and nuclear expression of HIF-1α (Figure 3C–F).

**Figure 4:**
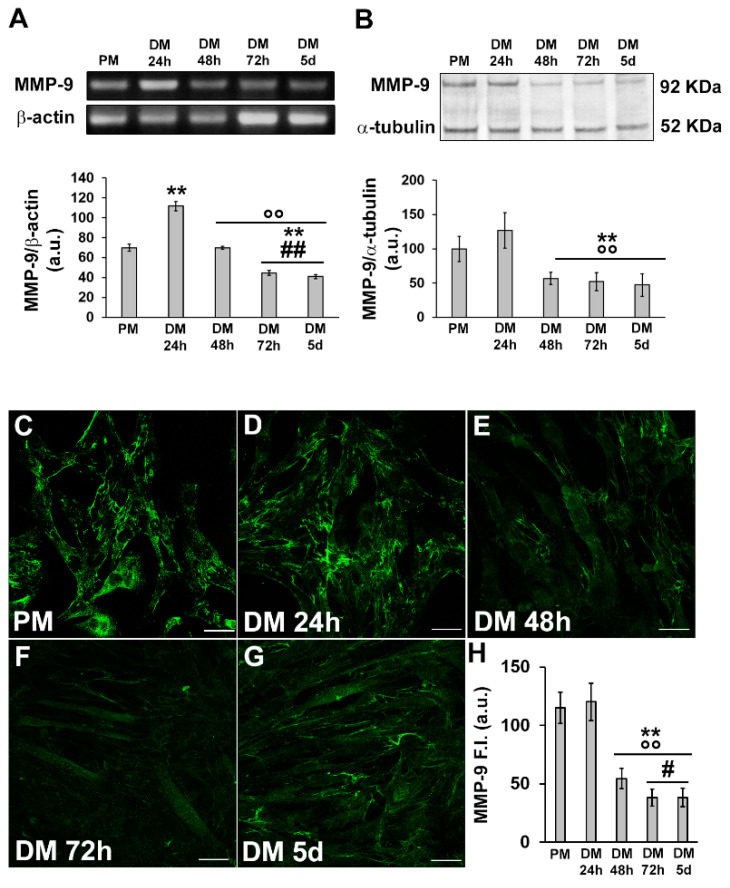
Evaluation of MMP-9 expression and cellular localization during C2C12 myoblast differentiation. The cells were cultured in the proliferation medium (PM) for 24 h or in the myogenic differentiation medium (DM) for different amounts of time (24, 48, 72 h and 5 d). (**A**) RT-PCR analysis. Representative agarose gel and bar charts showing the densitometric analysis of the bands normalized to β-actin. a.u.: arbitrary units. (**B**) Western blot analysis. Representative blot and bar charts showing the densitometric analysis of the bands normalized to α-tubulin. a.u.: arbitrary units. (**C**–**G**) Representative confocal fluorescence images of the cells immunostained with antibodies against MMP-9 (green). Scale bar: 25 µm. (**H**) Bar charts showing the densitometric analysis of the MMP-9 mean fluorescent signal intensity (F.I.) performed on digitized images in 10 regions of interest (ROI) of 100 μm^2^ for each confocal stack (10). a.u.: arbitrary units. Data are the results of at least three independent experiments performed in triplicate and are the mean ± SD. Statistical significance: ** *p* < 0.01 vs. PM; °° *p* < 0.01 vs. DM 24 h; # *p* < 0.05, ## *p* < 0.01 vs. DM 48 h (One-way ANOVA with post-hoc Tukey HSD).

In parallel, we analyzed the expression of MMP-9 at the mRNA and protein levels. We found that it mostly synchronized with that of HIF-1α, being higher in the early phase of myogenic differentiation and declining over the time (Figure 4A,B). Confocal immunofluorescence analysis revealed that MMP-9 was mainly localized in the cytoplasm along filamentous structures (Figure 4C–H).

**Figure 5:**
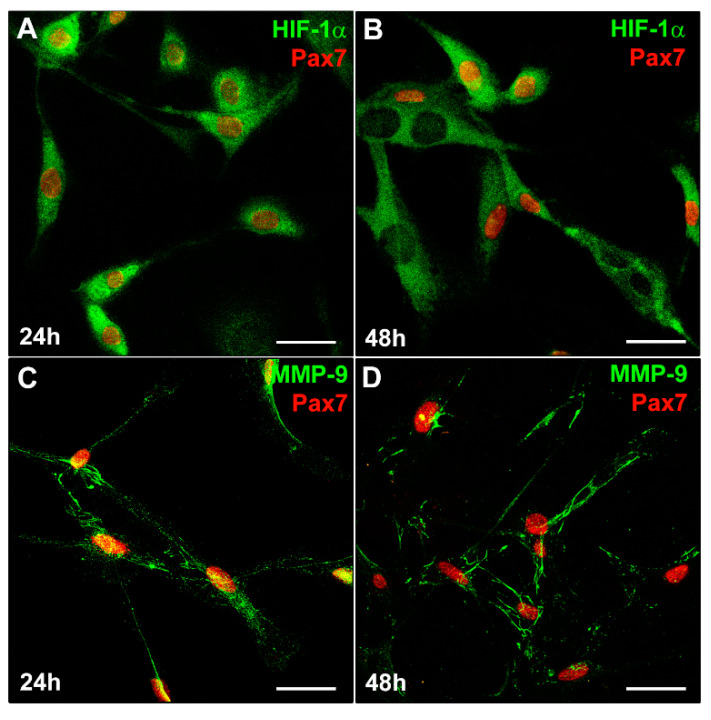
HIF-1α and MMP-9 expression in satellite cells (SCs). A murine SC-enriched culture was obtained from single skeletal EDL muscle fiber as reported in Material and Methods. The cells were cultured for 24 h and 48 h in their specific SC proliferation medium. Representative immunofluorescence confocal images of fixed SCs in the indicated experimental conditions, immunostained with (**A**,**B**) antibodies against Pax7 (red) and HIF-1α (green), (**C**,**D**) antibodies against Pax7 (red) and MMP-9 (green). Scale bar: 25 µm.

To increase the definition in our findings, we analyzed the expression of HIF-1α and MMP-9 in primary murine SCs that sprouted from single living myofibers isolated from EDL muscles (Figure 5).

Similarly to what we observed in C2C12 myoblasts (Figure 3C), Pax7^+^ SCs detected in the SC- enriched cultures, exhibited both nuclear and cytoplasmic expression of HIF-1α (Figure 5A) after 24 h of culture in their specific PM. After 48 h of culture (Figure 5B), multinucleated myotubes exhibited mainly a cytoplasmic expression of HIF-1α while showing, as expected, the downregulation of Pax7. Similarly to C2C12 myoblasts, SCs displayed a concomitant MMP-9 expression (Figure 5C,D).

### 3.2. HIF-1α and MMP-9 Are Required for Myogenic Commitment of Myoblasts and MMP-9 Is an Effector of HIF-1α

#### 3.2.1. Morphological and Biochemical Analyses

**Figure 6:**
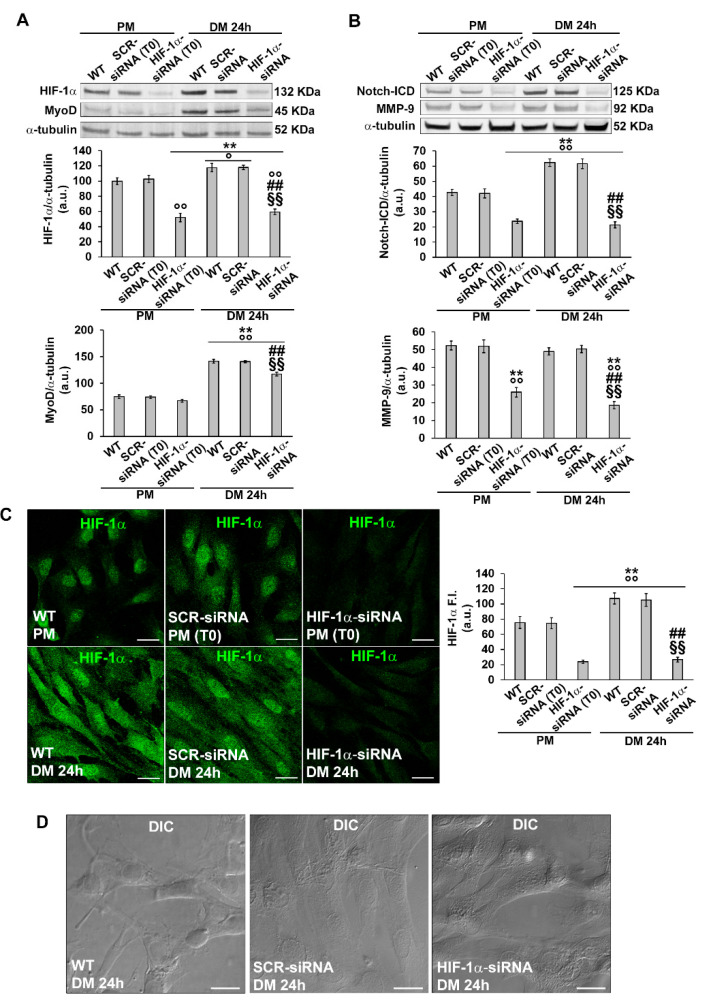
Evaluation of HIF-1α silencing on C2C12 myoblast MyoD, Notch-ICD and MMP-9 expression and morphology. The cells were transfected in the proliferation medium (PM) with either scrambled siRNA (SCR-siRNA) or HIF-1α-siRNA. After transfection—PM (T0)—the cells were induced to differentiate by shifting in the differentiation medium (DM) for 24 h. WT: wild type, not-transfected cells. (**A**,**B**) Western blot analysis. Representative blot and bar charts showing the densitometric analysis of the bands normalized to α-tubulin. a.u.: arbitrary units. (**C**) Representative confocal fluorescence images of the cells immunostained with antibodies against HIF-1α (green). Scale bar: 25 µm. Bar charts showing the densitometric analysis of the HIF-1α fluorescent signal intensity (F.I.) performed on digitized images in 10 regions of interest (ROI) of 100 μm^2^ for each confocal stack (10). a.u.: arbitrary units. Data shown are mean ± SD and represent the results of at least three independent experiments performed in triplicate. Significance of difference: ** *p* < 0.01 vs. WT PM (T0); ° *p* < 0.05, °° *p* < 0.01 vs. SCR-siRNA PM (T0); ## *p* < 0.01 vs. WT DM 24 h; §§ *p* < 0.01 vs. SCR-siRNA DM 24 h (One-way ANOVA with post-hoc Tukey HSD). (**D**) Representative differential interference contrast (DIC) images of fixed C2C12 cells in the indicated experimental conditions. Scale bar: 30 µm.

Based on the above findings concerning the temporal expression of HIF-1α and MMP-9, further analyses were focused on the early phase of cell differentiation (24 h). In particular, we evaluated the role of HIF-1α in the early stage of differentiation by silencing its expression by specific siRNA (PM T0) before culturing the cells in DM for 24 h (Figure 6A,C). As revealed by Western blot analysis, silenced cells exhibited a reduced expression of MyoD (Figure 6A) and of Notch-ICD (Figure 6B) compared to not-silenced cells (wild type, WT and SCR-siRNA).

**Figure 7:**
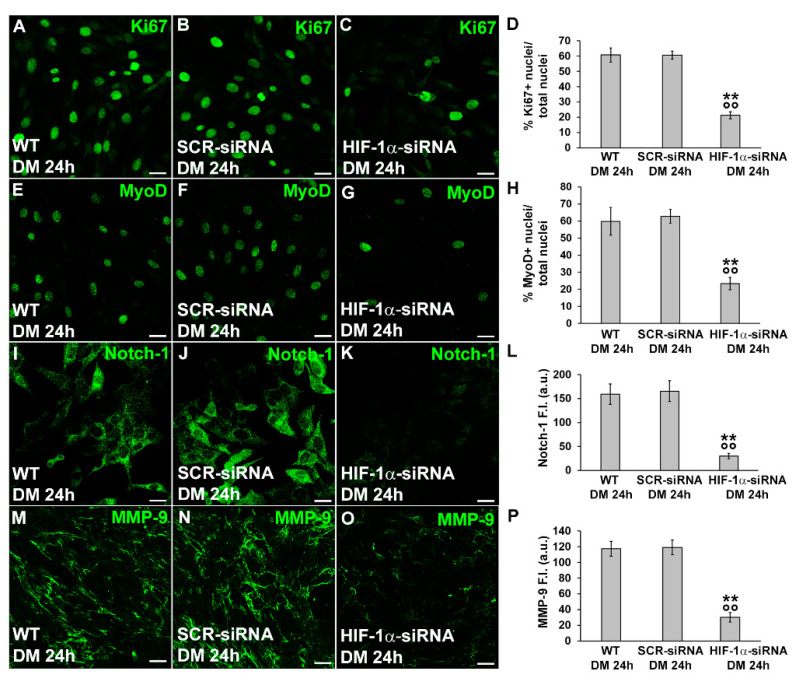
Evaluation of HIF-1α silencing on C2C12 myoblast Ki67, MyoD, Notch-1 and MMP-9 expression. The cells were transfected with either scrambled siRNA (SCR-siRNA) or HIF-1α-siRNA in the proliferation medium (PM). After transfection —PM (T0)—the cells were induced to differentiate by shifting in the differentiation medium (DM) for 24 h. WT: wild type, not-transfected cells. (**A**–**C**,**E**–**G**,**I**–**K**,**M**–**O**) Representative confocal fluorescence images of the cells immunostained with antibodies against the indicated markers (green). Scale bar: 25 µm. (**D**,**H**) Quantitative analyses of Ki67- and MyoD-positive nuclei expressed as the percentage of the total nuclei. (**L**,**P**) Bar charts showing densitometric analysis of the Notch-1 and MMP-9 fluorescent signal intensity (F.I.) performed on digitized images in 10 regions of interest (ROI) of 100 μm^2^ for each confocal stack (10). a.u.: arbitrary units. Data shown are mean ± SD and represent the results of at least three independent experiments performed in triplicate. Significance of difference: ** *p* < 0.01 vs. WT DM 24 h; °° *p* < 0.01 vs. SCR-siRNA DM 24 h (One-way ANOVA with post-hoc Tukey HSD).

Confocal immunofluorescence analysis, while corroborating Western Blotting results related to MyoD and Notch-1 (Figure 7E–L), also revealed a reduction in the percentage of Ki67 positive nuclei when the cells were silenced for HIF-1α expression (Figure 7A–D). Notably, as judged by DIC imaging, the silenced cells did not show those morphological features detectable in more advanced stages of differentiation such as alignment or fusion into myotubes (Figure 6D). The myogenin expression in myoblast induced to differentiate for 48 h revealed no significant difference between the cells silenced for HIF-1α and control cells (% myogenin nuclei/total nuclei: WT, 26.8 ± 4.9; SCR-siRNA, 24.6 ± 2.7; HIF-1α-siRNA, 25.2 ± 3.4; *p* > 0.05) suggesting that HIF-1α may not be involved in the regulation of such myogenic marker. However, differently from the control cells, silenced ones did not show a tendency to form polynucleated myotubes at this time point (Figure S1). These observations suggested that HIF-1α was required for cell commitment. Notably, silenced cells exhibited a reduced expression of MMP-9, suggesting that MMP-9 is a downstream effector of HIF-1α (Figure 6B and Figure 7M–P).

**Figure 8:**
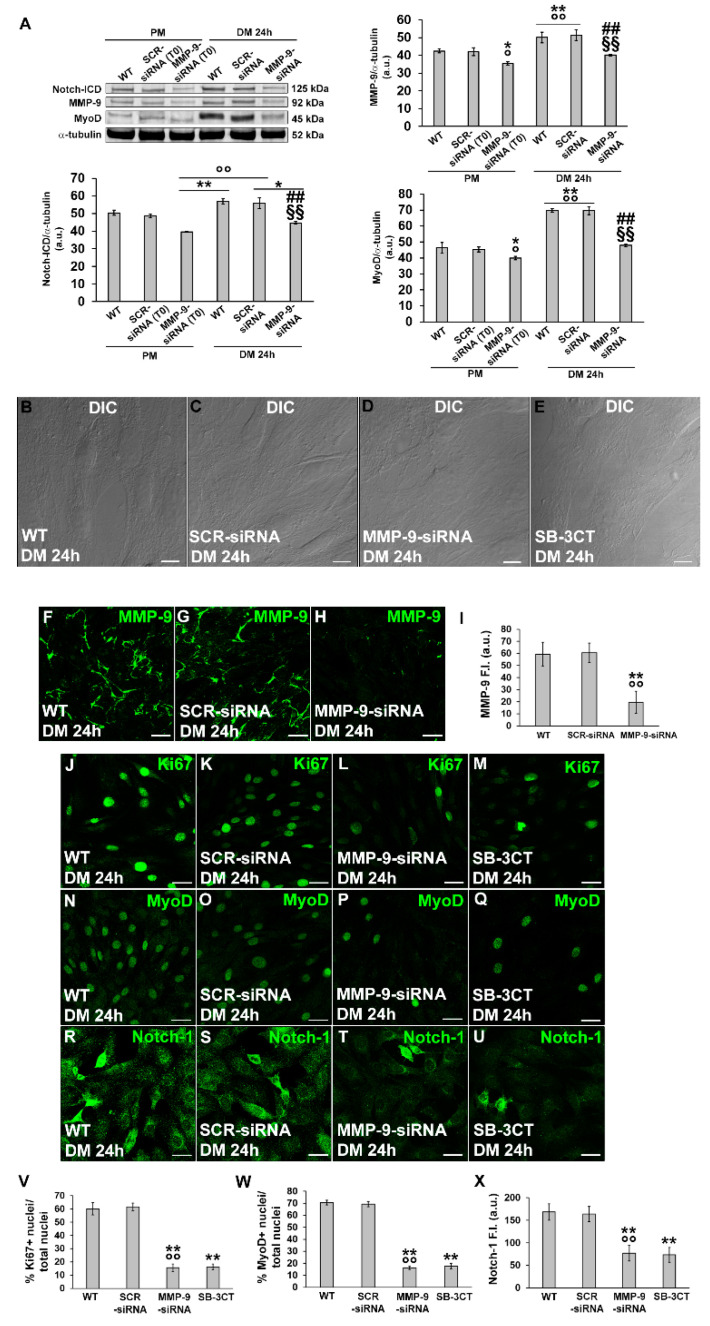
Evaluation of MMP-9 silencing or inhibition on C2C12 myoblast MyoD, Notch-1 and Ki67 expression and morphology. The cells were transfected in the proliferation medium (PM) with either scrambled siRNA (SCR-siRNA) or MMP-9-siRNA. After transfection—PM (T0)—the cells were shifted into the differentiation medium (DM) for 24 h to induce myogenic differentiation. In some experiments the cells were cultured in DM in the presence of the MMP-9 inhibitor SB-3CT (10 µM). WT: wild type, not-transfected or untreated cells. (**A**) Western blot analysis. Representative blot and bar charts showing the densitometric analysis of the bands normalized to α-tubulin. a.u.: arbitrary units. (**B**–**E**) Representative differential interference contrast (DIC) images of fixed C2C12 cells in the indicated experimental conditions. Scale bar: 12.5 µm. (**F**–**H**,**J**–**U**) Representative confocal fluorescence images of the cells immunostained with antibodies against the indicated markers (green). Scale bar: 25 µm. (**I**,**X**) Bar charts showing the densitometric analysis of the MMP-9 and Notch-1 fluorescent signal intensity (F.I.) performed on digitized images in 10 regions of interest (ROI) of 100 μm^2^ for each confocal stack (10). a.u.: arbitrary units. (**V**,**W**) Quantitative analyses of Ki67 and MyoD positive nuclei expressed as the percentage of the total nuclei, respectively. Data shown are mean ± SD and represent the results of at least three independent experiments performed in triplicate. Significance of difference: in (**A**): * *p* < 0.05, ** *p* < 0.01 vs. WT PM; ° *p* < 0.05, °° *p* < 0.01 vs. SCR-siRNA PM (T0); ## *p* < 0.01 vs. WT DM 24 h; §§ *p* < 0.01 vs. SCR-siRNA DM 24 h. In (**I**,**V**–**X**): ** *p* < 0.01 vs. WT; °° *p* < 0.01 vs. SCR-siRNA (One-way ANOVA with post-hoc Tukey HSD).

Finally, to address MMP-9’s role in the early phase of differentiation we performed experiments by inhibiting its expression with specific siRNA or its function with the pharmacological inhibitor SB-3CT (10 µM). We found that, after 24 h of culture in DM, C2C12 cells silenced for MMP-9 (Figure 8A,F–I) showed a reduction in Ki67 (Figure 8J–M,V), MyoD (Figure 8A,N–Q,W) and Notch-1 (Figure 8A,R–U,X), and exhibited morphological features of not differentiated cells that were to the cells silenced for HIF-1α (Figure 8B–D). Comparable results were obtained when the cells were cultured in DM in the presence of the MMP-9 inhibitor SB-3CT (Figure 8E,M,Q,U,V–X).

#### 3.2.2. Functional Electrophysiological Analyses

**Figure 9:**
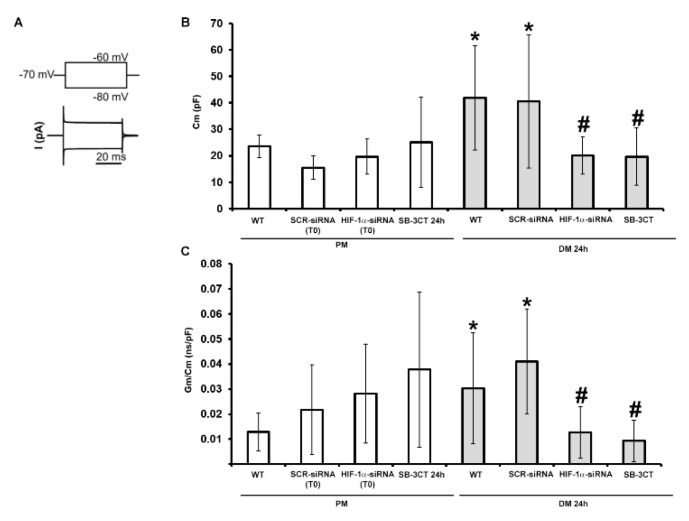
Electrophysiological analysis. The cells were transfected in the proliferation medium (PM) with scrambled siRNA (SCR-siRNA) or HIF-1α-siRNA. After transfection -PM (T0)- the cells were moved into the differentiation medium (DM) for 24 h to trigger myogenic differentiation. WT: wild type, not-transfected or untreated cells. In other experiments the cells were maintained in PM or DM with the MMP-9 inhibitor SB-3CT (10 µM) for 24 h. (**A**) Waveform of voltage-clamp simulation (top) and representative tracings of membrane passive currents (bottom) evoked in response to its application. (**B**) Cell capacitance (Cm, in pF). (**C**) Specific membrane conductance, Gm/Cm (in nS/pF). Data are mean ± SD. * *p* < 0.05 vs. WT PM, # *p* < 0.05 vs. WT DM 24 h (One way ANOVA followed by Bonferroni’s post hoc test).

To corroborate the results for the role of HIF-1α and MMP-9 on myogenic activation and commitment, we investigated some typical electrophysiological functional features of sarcolemma that better characterize an activated/committed myoblast (Figure 9).

Accordingly, we performed analyses on C2C12 cells grown in PM or in DM for 24 h. In parallel with the morphological evaluation, the cells were transfected in the proliferation medium (PM) with either scrambled siRNA (SCR-siRNA) or HIF-1α-siRNA and then differentiation was induced by shifting them into the differentiation medium (DM) for 24 h. Again, the not-transfected C2C12 cells are indicated as WT. In other experiments the cells were cultured in PM or DM in the presence of the MMP-9 inhibitor SB-3CT (10 µM) for 24 h. In particular, we first considered the cell membrane capacitance (Cm) values (Figure 9B) obtained by the analysis of the current records evoked by the step pulse protocol shown in Figure 9A (bottom and top, respectively). While these values did not significantly change in any conditions for cells grown in PM, we observed that Cm values recorded from WT C2C12 cells cultured in DM for 24 h were significantly higher than those measured from WT cells in PM. This suggests that, after 24 h of differentiation we could already observe significant variations ascribable to cell surface enlargement. SCR-siRNA cells showed Cm values similar to WT. In contrast, the silencing of HIF-1α expression determined a significant reduction in Cm values compared to those recorded in WT or SCR-siRNA cells in DM, and the results were not significantly different to those recorded in cells cultured in PM. The same effect was observed by blocking MMP-9 with the specific SB-3CT inhibitor. The specific membrane conductance Gm/Cm values (Figure 9C), in line with previous literature [49], tended to increase during the early phases of C2C12 cell differentiation, but did not show statistically significant differences when cells were grown in PM under any of the conditions tested. In contrast, WT C2C12 cells cultured in DM for 24 h showed a significant increase in the Gm/Cm compared to those cultured in PM. Such values again became similar to PM when HIF-1α was silenced or MMP-9 was inhibited. These experiments, achieved on cells in the early phase of differentiation (DM 24 h), strongly indicate the involvement of HIF-1α and MMP-9 in determining the increase in Cm and Gm/Cm, typically observed under the differentiation. The membrane passive properties’ values are listed in Table 2.

**Figure 10:**
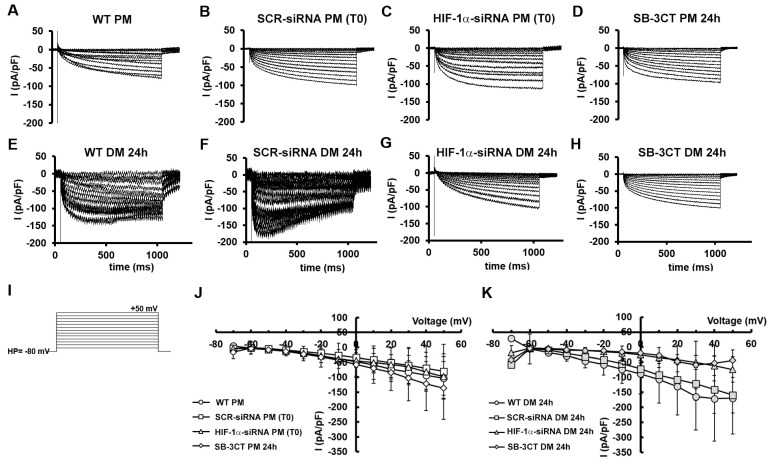
Ca^2+^ current evaluation in C2C12 cells. Representative time course of Ca^2+^ currents normalized for cell capacitance (in pA/pF) from (**A**) a wild type (WT) C2C12 cell cultured in proliferation medium PM or (**E**) differentiation medium DM for 24 h; (**B**) from a scrambled siRNA (SCR-siRNA) transfected cell cultured in PM (T0) or (**F**) in DM for 24 h; (**C**) from a HIF-1α-siRNA transfected cell cultured in PM (T0) or (**G**) in DM for 24 h; (**D**) from a cell treated with the MMP-9 inhibitor SB-3CT (10 µM) for 24 h in PM or (**H**) in DM. (**I**) Pulse protocol of stimulation used to evoke ion currents. (**J**) Overall I–V relation related to the four conditions for all the experiments performed in PM (WT PM, n = 7, open circles; SCR-siRNA PM (T0) n = 4, open squares; HIF-1α-siRNA PM (T0) n = 4, open triangles; SB-3CT PM 24 h n = 6, open diamonds) and (**K**) in DM (WT DM 24 h n = 6, gray circles; SCR-siRNA DM 24 h n = 2, gray squares; HIF-1α-siRNA DM 24 h n = 3, gray triangles; SB-3CT DM 24 h n = 7, gray diamonds). Data are mean ± SD. (*p* > 0.05 two-way ANOVA).

Finally, by applying a suitable voltage pulse protocol of stimulation (Figure 10I), we evaluated the possible transmembrane ion current flux elicited by the activation of the voltage-operated Ca^2+^ channels (I_Ca_), as a typical feature of skeletal muscle differentiation. WT C2C12 myoblasts grown in PM showed an inward, almost not-inactivating current (Figure 10A), without the clear presence of a voltage threshold. Notably, when cells were kept in PM, we observed analogous inward current time courses in all of the conditions analyzed (Figure 10A–D).

The I-V plots related to all the experiments achieved on the cells grown in PM (Figure 10J) exhibited a current amplitude increase that was approximately linear with the applied voltage step increment, suggesting an almost not voltage-dependent ion entry. Moreover, the mean current values were almost overlapping despite the different treatments. In contrast, WT cells cultured in DM for 24 h (Figure 10E) typically showed an inward current with a larger amplitude and a sort of inactivation at higher voltage steps, resembling the I_Ca_ time course of a more differentiated skeletal muscle cell. As expected, a similar time course was observed in SCR-siRNA (Figure 10F). Nonetheless, when HIF-1α was silenced, we observed current records (Figure 10G) with a time course quite different from those observed in WT cells in DM, but very similar to those recorded from cells in PM (Figure 10A) that varied linearly with the voltage step applied, as shown by the I-V plot in Figure 10K (triangles). This phenomenon was pretty similar to that evoked in WT myoblasts in PM, suggesting that, although in DM, HIF-1α-silenced cells still maintain the same ion channel profile/features shown by WT cells in PM. Finally, when MMP-9 was inhibited by SB-3CT (Figure 10H) the current time course recorded from cells in DM was quite different from the WT counterpart in the same medium, again resembling that observed in C2C12 WT in PM. The I-V plot also supported this outcome (Figure 10K, diamonds) since data related to HIF-1α silencing and MMP-9 inhibitor were almost superimposed. In contrast, the mean values of the current amplitudes recorded from WT and SCR-siRNA tended to be higher and more similar. This result may further indicate that HIF-1α and MMP-9 have a role in driving ion channels expression during the early differentiation phase of C2C12 myoblasts.

## 4. Discussion

The current study provides experimental evidence that HIF-1α plays a critical function during myoblast differentiation in vitro that is not strictly related to hypoxic conditions. In fact, we have uncovered a previously unrecognized relationship between HIF-1α and MMP-9 in myoblasts induced to differentiate under normoxia conditions. In particular, we have evinced the essential role of such an axis in the very early phases of normoxic myogenic progression, namely activation and commitment. These conclusions arise from the following observations.

First, morphological and biochemical analyses showed the different spatial and temporal expression of HIF-1α subunit in the C2C12 myoblasts undergoing differentiation. In particular, HIF-1α expression increased after 24 h of culture in differentiating condition (DM) compared to those observed in the cells in growing condition (PM) and declined with differentiating time. Interestingly, HIF-1α expression is synchronized with that of the myogenic activation marker MyoD and with Notch-1, a crucial determinant of myoblast activation and proliferation. Proliferating committed mononuclear myoblasts exhibited a more marked HIF-1α expression at the nuclear level as compared to the more elongated cells and polynucleated myotubes. The expression pattern of HIF-1α was confirmed in murine primary SCs. Conversely, Wagatsuma et al., 2011 [29], found that C2C12 cells showed a decrease in HIF-1α protein expression when moving from a growth to a differentiation medium, whereas Ono et al., 2006 [21], showed that the HIF-1α subunit was hardly identified in undifferentiated C2C12 myoblasts cultured in growth medium. This discordance can be attributed to nonidentical experimental cell culture conditions. However, in accordance with our findings and other previous research [19,28], these papers [21,29] demonstrated the nuclear expression of HIF-1α in the early phase (induction) of C2C12 cell myogenesis. In myoblasts the nuclear HIF-1α expression may be consistent with the functional induction of the genes encoding glycolytic enzymes required to satisfy the cell energy demand in the early phases of the myogenic differentiation process [7,8,9,19,50,51,52]. Along these lines, we have confirmed, in our experimental myogenesis model, that the cells undergo metabolic reprogramming during the differentiation process; in fact, proliferating myoblasts mostly use glycolysis to produce energy whereas myotubes show a more developed aerobic capacity, as assessed by the analyses of the expression of LDH-A and SDH-B. Moreover, during the differentiation phase we have observed an increase in the expression of PGC-1α that has been demonstrated to regulate LDH by reducing LDH-A mRNA transcription and the enzymatic activity that converts pyruvate to lactate [53]. In addition, PGC-1α has been acknowledged as a crucial modulator of mitochondrial biogenesis. Indeed, a clear consensus is evident in the literature on the occurrence of mitochondrial biogenesis during myogenic differentiation so as to sustain the energy demand required for myotube formation and successively polynucleated long myofibers [54]. Moreover, keeping in mind the role of vascular endothelial growth factor (VEGF)/VEGF receptor-mediated signaling in myogenic differentiation [47,55,56,57] and the well-known cross-talk between HIF-1α and such signaling [19,58,59,60], we may also postulate the possibility that HIF-1α is physiologically involved in myoblast activation and proliferation by modulating VEGF signaling. Different from the protein expression, we have found that HIF-1α mRNA appeared to be constantly expressed in C2C12 cells either under growth or differentiating steps according to previous data [19,21,28,29]. Our results seem to indicate that the differences in protein level are not due to a different mRNA expression and suggest instead that the regulation of HIF-1α expression and activity during myogenic differentiation in vitro occurred at post-transcriptional level, possibly including protein transduction and stabilization/degradation, nuclear localization and transactivation.

The hypoxia-independent expression of HIF-1α in myoblastic cells during differentiation is not surprising, as demonstrated in previous research [21,22,29]. This observation is also in line with the growing evidence demonstrating that HIF-1α is involved in diverse biological functions that need its activation with normal oxygen levels. The exact reason why HIF-1α escapes proteasome degradation in our cell model is not explained. However, non-hypoxic activators of HIF-1α have been described including, among others, nitric oxide, NO [61,62], which has been demonstrated to be produced by C2C12 myoblasts, peaking within 24 h after inducing differentiation [63]. Moreover, the stability of HIF-1α in skeletal muscle cells depends on different regulators, including not only the oxygen sensors named prolyl hydroxylases PHDs that guide the oxygen-dependent HIF-1α degradation, but also the heat shock protein 90 (Hsp90) whose content and expression is independent of oxygen concentration. In particular, it has been observed that Hsp90 protects HIF-1α from proteasome breakdown promoting its accumulation during in vitro differentiation of myoblastic cells under normoxia [21,22]. A PCG-1α dependent stabilization of HIF-1α has also been proposed in skeletal muscle cells [64].

Furthermore, the observed HIF-1α expression in the cytoplasm may suggest an unconventional function of HIF-1α, as a signaling regulator distinct from its canonical activity as a transcriptional factor [65,66,67]. The specific mechanisms modulating HIF-1α expression need further exploration.

The possible critical role of HIF-1α during the early phases of myogenesis under normoxia, suggested by our first results, has been then confirmed by experiments in which HIF-1α gene expression was silenced by specific siRNA. Indeed, silenced cells cultured in myogenic differentiation medium showed a reduction of the activation markers tested, namely Ki67, Notch-1 and MyoD. Keeping in mind that, during the process of myoblast differentiation, MyoD expression increases at first and then decreases, then [68], as confirmed in our experimental model, our results might also suggest a more differentiated state of the silenced cells. In other words, the possibility that HIF-1α could function to counteract differentiation might also be considered. However, morphological analysis uncovered that silenced cells did not show at this time point features of more differentiated cells such as elongation, alignment or fusion into multinucleated myotubes. However, we found that HIF-1α knockdown did not hamper myogenin expression in cells induced to differentiate for 48 h in agreement with previous reports [12,28], indicating that HIF-1α may not be involved in the regulation of such a myogenic marker under normoxia.

All these observations led us to suggest that HIF-1α is required for cell activation/commitment.

Our findings are consistent with previous data demonstrating that, in C2C12, myoblasts HIF-1α knockdown—mediated by siRNA—results in the inhibition of myotube formation [21]. They are also in line with data from Cirilli et al. [15] showing that the pharmacological activation of HIF-1α, following treatment with some PHDs inhibitors, promoted C2C12 myoblast differentiation. In contrast, it has been shown that the constitutive expression of active HIF-1α in C2C12 myoblasts did not induce faster differentiation like the ectopic expression of DEC1/Stra13, a target gene of HIF-1α, which did not prevent C2C12 myotube formation under either hypoxia or normoxia [69], supporting the notion that HIF-1α is not directly involved in postnatal myogenesis. Nevertheless, all these observations may suggest the requirement of a coordinated temporal modulation of HIF-1α and thus it cannot be excluded that: (i) HIF-1α functionality may be critical just for some steps of the myogenic process, (ii) HIF-1 may act differently during the differentiation period. Therefore, the above cited papers are not in contrast with the findings of the current research. However, the role of HIF-1α in the late phase of myogenesis in our model remains to be investigated deeper.

A novel finding from this study is the demonstration that MMP-9 is a downstream effector of HIF-1α and that the HIF-1α/MMP-9 signaling pathway is involved in myoblast activation. Indeed, we found that MMP-9 expression synchronized with that of HIF-1α showing higher level after 24 h of differentiation, and that myoblasts silenced for HIF-1α exhibited a reduction in MMP-9 expression. Of note, the gene silencing of MMP-9 or its pharmacological inhibition caused a decrease in the activation markers of myogenesis similarly to HIF-1α silencing, thus suggesting an unconventional intracellular action of such proteases. In line with this, previous research conducted by our group unraveled that bone marrow mesenchymal stromal cell secretome was able to increase MMP-9 expression in murine myoblasts and that this event was accompanied by an increase in cell mobilization, proliferation and activation [38]. Consistent with our findings, an earlier study demonstrated that the inhibition of MMP-9 (with doxycycline and anti-MMP-9) delayed in vitro proliferation and differentiation of satellite cell-derived myoblasts from rat Soleus muscle [70]. The same research group documented that proliferating rat myoblasts exhibited a nuclear expression of MMP-9 and that MMP-9 activity was required for proper cell cycle progression, since its inhibition affected myoblast proliferation [39,41]. In contrast to Zimowska et al., we did not observe a nuclear localization of the protein suggesting that in our experimental model MMP-9 is not directly involved in nuclear events. Based on the observed distribution of MMP-9 that appeared linearly organized along filamentous structures, most likely cytoskeletal ones, we may hypothesize the occurrence of an MMP-9-dependent cytoskeletal rearrangement required for driving myoblast activation and differentiation [49,71,72].

A strength of this research is that the outcomes from biomolecular, biochemical and morphological analyses were robustly supported by the functional electrophysiological recordings. In particular, the evaluation of the passive membrane properties of the committed myoblasts focused on those parameters supposed to be suggestive of a more differentiated/mature phenotype. Accordingly, the cell capacitance (Cm) value, usually related to the cell surface, is expected to increase during differentiation [73,74]. This was actually observed in our cells induced to differentiate, but when HIF-1α expression was silenced or MMP-9 was inhibited, its values turned out to be similar (not statistically different) to those measured in proliferating (not differentiating) myoblasts. In addition, our preliminary results on resting membrane potential confirmed these indications showing an expected hyperpolarization in myotubes as previously observed [46]. Analogous observations could be made for the analysis of the specific conductance (Gm/Cm) that can be considered another index of differentiation [75]. The Gm/Cm values significantly increased in cells moving from the proliferation to the differentiation medium indicating an increased membrane permeability. Notably, this Gm/Cm increase was not observed in cells where HIF-1α was silenced or MMP-9 inhibited. These findings may be correlated with the possible involvement of HIF-1α and MMP-9 in the regulation of protein fusion machines including Myomaker (Mymk) and Myomerger/myomixer (Mymx), that control different steps of membrane remodeling during myoblast fusion pathway. Indeed, it has been reported that myoblasts undergoing differentiation may establish membrane fusion connections forming early fusion intermediates that may or may not advance to complete fusion in multinucleated myotubes [76]. While Mymk is essential for fusion initiation and formation of hemifusion intermediates, Mymx drives the subsequent step of fusion with pore formation. In fact, Mymx renders membranes susceptible to permeabilization, leading to the opening of fusion pores in the hemifusion intermediate which is likely located at the junction of concave-shaped lipid monolayers that come together at the rim of the hemifusion intermediate [77,78]. Based on these considerations, we can speculate a role for HIF-1α and MMP-9 in modulating membrane permeability and specific membrane conductance via the regulation of protein fusion machines. This remains an open question that needs to be further studied in dedicated future research. Finally, we intended to estimate the ion current flowing through voltage-activated Ca^2+^ channels. This, in fact, can be considered as a typical feature of skeletal muscle differentiation due to its crucial role in excitation–contraction (EC) coupling in mature skeletal myofibers [79]. Although the structure of L-type calcium channels in skeletal muscle consists of the pore forming α1 subunit, and the α2δ, β and γ subunits, interestingly, these subunits are usually not expressed at the same moment during differentiation of myotubes in vitro [80,81,82]. This fact may undoubtedly hamper the early acquisition of fully functional voltage-dependent properties and their consequent clear revelation by electrophysiological techniques. Indeed, in our study we could observe that untreated myoblasts (WT) grown in PM usually showed an inward not-inactivating current suggesting an almost not voltage-dependent ion influx. On the contrary, although 24 h might be a very early differentiation stage, myoblasts cultured in DM exhibited an inward current with an increased amplitude and a type of inactivation at higher voltage steps, resembling the I_Ca_ time course of a more differentiated skeletal muscle cell. Again, the current recorded in cells silenced for HIF-1α was very similar to that evoked in WT myoblasts in PM; although, in DM, HIF-1α-silenced cells maintain the same ion channel profile/features shown by WT in PM. A similar result was observed when MMP-9 was inhibited by SB-3CT. This may suggest that HIF-1α and MMP-9 have a role in C2C12 myoblasts driving Ca^2+^ channels’ expression/assembly during the early differentiation phase.

This hypothetical role of HIF-1α may be supported by previously published data, even though these were achieved in a different muscle tissue, namely in pulmonary arterial smooth muscle cells [83]. The authors reported that HIF-1α plays a key role in Ca^2+^ homeostasis, specifically in modulating the expression of transient receptor potential channels (TRPCs), thus contributing to pulmonary vascular remodeling.

## 5. Conclusions

In conclusion, our results highlight that HIF-1α plays a key role in normoxically differentiating skeletal myoblasts in vitro and corroborate the notion that MMP-9 is a downstream effector of this factor in myoblasts. Here, we demonstrate, for the first time to our knowledge, that the HIF-1α/MMP-9 axis is required for myoblast activation and commitment steps, thus broadening the understanding of the myogenic machinery.

Whether MMP-9 represents a direct or indirect target of HIF-1α in our cell model needs to be proved. The possibility that HIF-1α regulates MMP-9 by acting on tissue inhibitors of metalloproteinases (TIMPs) cannot be excluded, particularly when considering that TIMP-1 and 2 are expressed in C2C12 myoblasts [38] and that a crosstalk between TIMPs and HIF-1α has been demonstrated in other cell types [84,85,86,87,88,89]. Moreover, it has been reported that MMP-13 plays a pivotal role in the MMP activation cascade and, specifically, it can activate MMP-9 [90]. Keeping in mind this relationship and previously published data showing a determinant role of MMP-13 on migration and differentiation of C2C12 myoblasts (although with a little effect on cell proliferation or fusion) [91], it could be of interest to consider MMP-13 as an effector of HIF-1α, particularly in the later phases of differentiation.

A deeper insight into the molecular pathways that drive and coordinate skeletal myogenesis is essential to improve the comprehension of skeletal muscle disorders and pathologies. In this regard, these results may provide cues for the identification of novel smart targets that could be exploited to boost the intrinsic regenerative potential of muscle tissue, thus contributing to increasing the therapeutic proposals in muscle disease management. Indeed, SCs are notoriously scarce in muscle tissue, representing 2–10% of the total myonuclei [4]. Moreover, in case of severe or extended damage or in some pathological muscle condition their function may also be compromised and the inefficient muscle regeneration replaces the muscle tissue with a fibrotic not contractile tissue. In such a context, strategies aimed at improving endogenous regenerative potential including SC activity may offer promising perspectives.

## Figures and Tables

**Table 1:** Primary antibodies employed in immunofluorescence (IF) and Western blotting (WB) analyses.

Primary Antibody Name	Company	Code	Dilution
anti-Ki67 rabbit polyclonal	Abcam, Cambridge, UK	ab15580	1:100 (IF)
anti-Notch-1 rabbit monoclonal	Abcam	ab52627	1:100 (IF) 1:1000 (WB)
anti-MyoD (M-318) rabbit polyclonal	Santa Cruz Biotechnology	sc-760	1:50 (IF) 1:500 (WB)
anti-myogenin (F5D) mouse monoclonal	Santa Cruz Biotechnology	sc-12732	1:50 (IF)
anti-α-sarcomeric actin mouse monoclonal	DakoCytomation, Carpinteria, CA, USA	M 0874	1:100 (IF)
anti- peroxisome proliferator-activated receptor-gamma coactivator (PGC)-1α mouse monoclonal	Santa Cruz Biotechnology	sc-518025	1:100 (IF)
anti-HIF-1α rabbit polyclonal	Santa Cruz Biotechnology	sc-10790	1:100 (IF) 1:1000 (WB)
anti-MMP-9 rabbit polyclonal	Bioss Antibodies, Woburn, MA, USA	bs-0397R	1:100 (IF) 1:1000 (WB)
anti-Pax7 mouse monoclonal	Santa Cruz Biotechnology	sc-81648	1:100 (IF)
anti-succinate dehydrogenase complex iron sulfur subunit-B (SDH-B) rabbit polyclonal	Sigma-Aldrich	SAB2102103	1:1000 (WB)
anti-lactate dehydrogenase (LDH)-A (E-9) mouse monoclonal	Santa Cruz Biotechnology	sc-137243	1:1000 (WB)
anti-α-tubulin rabbit polyclonal	GeneTex, Prodotti Gianni, Milano, Italy	GTX112141	1:1000 (WB)

**Table 2:** C2C12 cells passive membrane properties. The cells were treated and cultured as indicated in Figure 9. At least three independent C2C12 cell culture preparations were analyzed. n: number of the cells. Data are mean ± SD. * *p* < 0.05 vs. WT PM; # *p* < 0.05 vs. WT DM 24 h (One way ANOVA followed by Bonferroni’s post hoc test).

Condition	Cm (pF)	Gm/Cm (nS/pF)
WT PM	23.56 ± 4.20 n = 5	1.29 × 10^−2^ ± 0.77 × 10^−2^ n = 10
SCR-siRNA PM (T0)	15.51 ± 4.43 n = 3	2.18 × 10^−2^ ± 1.79 × 10^−2^ n = 4
HIF-1α-siRNA PM (T0)	19.65 ± 6.68 n = 4	2.82 × 10^−2^ ± 1.97 × 10^−2^ n = 4
SB-3CT PM 24 h	25.08 ± 17.06 n = 6	3.78 × 10^−2^ ± 3.10 × 10^−2^ n = 5
WT DM 24 h	41.87 ± 19.62 * n = 10	3.04 × 10^−2^ ± 2.21 × 10^−2^ * n = 10
SCR-siRNA DM 24 h	40.58 ± 25.22 * n = 7	4.11 × 10^−2^ ± 2,09 × 10^−2^ * n = 6
HIF-1α-siRNA DM 24 h	20.08 ± 7.02 # n = 5	1.27 × 10^−2^ ± 1.03 × 10^−2^ # n = 11
SB-3CT DM 24 h	19.68 ± 10.81 # n = 5	0.93 × 10^−2^ ± 0.82 × 10^−2^ # n = 4

## Data Availability

Data are available from the corresponding author upon reasonable request.

## Supplementary Materials

The following supporting information can be downloaded at: https://www.mdpi.com/article/10.3390/cells12242851/s1, Figure S1: Evaluation of HIF-1α silencing on C2C12 myoblast myogenin expression.

## References

[1] Sousa-Victor P., García-Prat L., Muñoz-Cánoves P. (2022). Control of satellite cell function in muscle regeneration and its disruption in ageing. Nat. Rev. Mol. Cell Biol..

[2] Feige P., Brun C.E., Ritso M., Rudnicki M.A. (2018). Orienting Muscle Stem Cells for Regeneration in Homeostasis, Aging, and Disease. Cell Stem Cell.

[3] Johnson A.L., Kamal M., Parise G. (2023). The Role of Supporting Cell Populations in Satellite Cell Mediated Muscle Repair. Cells.

[4] Forcina L., Cosentino M., Musarò A. (2020). Mechanisms Regulating Muscle Regeneration: Insights into the Interrelated and Time-Dependent Phases of Tissue Healing. Cells.

[5] Manetti M., Tani A., Rosa I., Chellini F., Squecco R., Idrizaj E., Zecchi-Orlandini S., Ibba-Manneschi L., Sassoli C. (2019). Morphological evidence for telocytes as stromal cells supporting satellite cell activation in eccentric contraction-induced skeletal muscle injury. Sci. Rep..

[6] Collins C.A., Olsen I., Zammit P.S., Heslop L., Petrie A., Partridge T.A., Morgan J.E. (2005). Stem Cell Function, Self-Renewal, and Behavioral Heterogeneity of Cells from the Adult Muscle Satellite Cell Niche. Cell.

[7] Takeda K., Takemasa T., Fujita R. (2023). High Throughput Screening of Mitochondrial Bioenergetics in Myoblasts and Differentiated Myotubes. Methods Mol. Biol..

[8] Singh V. (2021). Intracellular metabolic reprogramming mediated by micro-RNAs in differentiating and proliferating cells under non-diseased conditions. Mol. Biol. Rep..

[9] Bhattacharya D., Scimè A. (2020). Mitochondrial Function in Muscle Stem Cell Fates. Front. Cell Dev. Biol..

[10] Cirillo F., Mangiavini L., La Rocca P., Piccoli M., Ghiroldi A., Rota P., Tarantino A., Canciani B., Coviello S., Messina C. (2022). Human Sarcopenic Myoblasts Can Be Rescued by Pharmacological Reactivation of HIF-1α. Int. J. Mol. Sci..

[11] Salekeen R., Kyba M. (2022). Not young but still immature: A HIF-1α-mediated maturation checkpoint in regenerating muscle. J. Clin. Investig..

[12] Lu Y., Mao J., Han X., Zhang W., Li Y., Liu Y., Li Q. (2021). Downregulated hypoxia-inducible factor 1α improves myoblast differentiation under hypoxic condition in mouse genioglossus. Mol. Cell. Biochem..

[13] Nguyen T.H., Conotte S., Belayew A., Declèves A.E., Legrand A., Tassin A. (2021). Hypoxia and Hypoxia-Inducible Factor Signaling in Muscular Dystrophies: Cause and Consequences. Int. J. Mol. Sci..

[14] Pircher T., Wackerhage H., Aszodi A., Kammerlander C., Böcker W., Saller M.M. (2021). Hypoxic Signaling in Skeletal Muscle Maintenance and Regeneration: A Systematic Review. Front. Physiol..

[15] Cirillo F., Resmini G., Angelino E., Ferrara M., Tarantino A., Piccoli M., Rota P., Ghiroldi A., Monasky M.M., Ciconte G. (2020). HIF-1α Directly Controls WNT7A Expression During Myogenesis. Front. Cell Dev. Biol..

[16] Sinha K.M., Tseng C., Guo P., Lu A., Pan H., Gao X., Andrews R., Eltzschig H., Huard J. (2019). Hypoxia-inducible factor 1α (HIF-1α) is a major determinant in the enhanced function of muscle-derived progenitors from MRL/MpJ mice. FASEB J..

[17] Cirillo F., Resmini G., Ghiroldi A., Piccoli M., Bergante S., Tettamanti G., Anastasia L. (2017). Activation of the hypoxia-inducible factor 1α promotes myogenesis through the noncanonical Wnt pathway, leading to hypertrophic myotubes. FASEB J..

[18] Yang X., Yang S., Wang C., Kuang S. (2017). The hypoxia-inducible factors HIF1α and HIF2α are dispensable for embryonic muscle development but essential for postnatal muscle regeneration. J. Biol. Chem..

[19] Dehne N., Kerkweg U., Otto T., Fandrey J. (2007). The HIF-1 response to simulated ischemia in mouse skeletal muscle cells neither enhances glycolysis nor prevents myotube cell death. Am. J. Physiol. Regul. Integr. Comp. Physiol..

[20] Li X., Zhu L., Chen X., Fan M. (2007). Effects of hypoxia on proliferation and differentiation of myoblasts. Med. Hypotheses.

[21] Ono Y., Sensui H., Sakamoto Y., Nagatomi R. (2006). Knockdown of hypoxia-inducible factor-1alpha by siRNA inhibits C2C12 myoblast differentiation. J. Cell. Biochem..

[22] Kubis H.P., Hanke N., Scheibe R.J., Gros G. (2005). Accumulation and nuclear import of HIF1 alpha during high and low oxygen concentration in skeletal muscle cells in primary culture. Biochim. Biophys. Acta.

[23] Kang J.S., Kim D., Rhee J., Seo J.Y., Park I., Kim J.H., Lee D., Lee W., Kim Y.L., Yoo K. (2023). Baf155 regulates skeletal muscle metabolism via HIF-1a signaling. PLoS Biol..

[24] Hao T., Liu Y.H., Li Y.Y., Lu Y., Xu H.Y. (2017). A Transcriptomic Analysis of Physiological Significance of Hypoxia-inducible Factor-1α in Myogenesis and Carbohydrate Metabolism of Genioglossus in Mice. Chin. Med. J..

[25] Keith B., Simon M.C. (2007). Hypoxia-inducible factors, stem cells, and cancer. Cell.

[26] Kitakaze T., Sugihira T., Kameyama H., Maruchi A., Kobayashi Y., Harada N., Yamaji R. (2022). Carotenoid transporter CD36 expression depends on hypoxia-inducible factor-1α in mouse soleus muscles. J. Clin. Biochem. Nutr..

[27] Settelmeier S., Schreiber T., Mäki J., Byts N., Koivunen P., Myllyharju J., Fandrey J., Winning S. (2020). Prolyl hydroxylase domain 2 reduction enhances skeletal muscle tissue regeneration after soft tissue trauma in mice. PLoS ONE.

[28] Cicchillitti L., Di Stefano V., Isaia E., Crimaldi L., Fasanaro P., Ambrosino V., Antonini A., Capogrossi M.C., Gaetano C., Piaggio G. (2012). Hypoxia-inducible factor 1-α induces miR-210 in normoxic differentiating myoblasts. J. Biol. Chem..

[29] Wagatsuma A., Kotake N., Yamada S. (2011). Spatial and temporal expression of hypoxia-inducible factor-1α during myogenesis in vivo and in vitro. Mol. Cell. Biochem..

[30] Mounier R., Pedersen B.K., Plomgaard P. (2010). Muscle-specific expression of hypoxia-inducible factor in human skeletal muscle. Exp. Physiol..

[31] Stroka D.M., Burkhardt T., Desbaillets I., Wenger R.H., Neil D.A., Bauer C., Gassmann M., Candinas D. (2001). HIF-1 is expressed in normoxic tissue and displays an organ-specific regulation under systemic hypoxia. FASEB J..

[32] Majmundar A.J., Lee D.S., Skuli N., Mesquita R.C., Kim M.N., Yodh A.G., Nguyen-McCarty M., Li B., Simon M.C. (2015). HIF modulation of Wnt signaling regulates skeletal myogenesis in vivo. Development.

[33] Gustafsson M.V., Zheng X., Pereira T., Gradin K., Jin S., Lundkvist J., Ruas J.L., Poellinger L., Lendahl U., Bondesson M. (2005). Hypoxia requires notch signaling to maintain the undifferentiated cell state. Dev. Cell.

[34] Li H., Huang H., Cui Y., Li W., Zhang S., Chen Y. (2021). Study on the Mechanism of Capillary Leakage Caused by Hypoxia-Inducible Factor-1α through Inducing High Expression of Matrix Metalloproteinase-9. J. Oncol..

[35] Li Y.Y., Zheng Y.L. (2017). Hypoxia promotes invasion of retinoblastoma cells in vitro by upregulating HIF-1α/MMP9 signaling pathway. Eur. Rev. Med. Pharmacol. Sci..

[36] Choi J.Y., Jang Y.S., Min S.Y., Song J.Y. (2011). Overexpression of MMP-9 and HIF-1α in Breast Cancer Cells under Hypoxic Conditions. J. Breast Cancer.

[37] Du R., Lu K.V., Petritsch C., Liu P., Ganss R., Passegué E., Song H., Vandenberg S., Johnson R.S., Werb Z. (2008). HIF1alpha induces the recruitment of bone marrow-derived vascular modulatory cells to regulate tumor angiogenesis and invasion. Cancer Cell.

[38] Sassoli C., Nosi D., Tani A., Chellini F., Mazzanti B., Quercioli F., Zecchi-Orlandini S., Formigli L. (2014). Defining the role of mesenchymal stromal cells on the regulation of matrix metalloproteinases in skeletal muscle cells. Exp. Cell Res..

[39] Nowak E., Gawor M., Ciemerych M.A., Zimowska M. (2018). Silencing of gelatinase expression delays myoblast differentiation in vitro. Cell Biol. Int..

[40] Rebalka I.A., Monaco C.M.F., Varah N.E., Berger T., D’souza D.M., Zhou S., Mak T.W., Hawke T.J. (2018). Loss of the adipokine lipocalin-2 impairs satellite cell activation and skeletal muscle regeneration. Am. J. Physiol. Cell Physiol..

[41] Zimowska M., Swierczynska M., Ciemerych M.A. (2013). Nuclear MMP-9 role in the regulation of rat skeletal myoblasts proliferation. Biol. Cell.

[42] Squecco R., Chellini F., Idrizaj E., Tani A., Garella R., Pancani S., Pavan P., Bambi F., Zecchi-Orlandini S., Sassoli C. (2020). Platelet-Rich Plasma Modulates Gap Junction Functionality and Connexin 43 and 26 Expression During TGF-β1-Induced Fibroblast to Myofibroblast Transition: Clues for Counteracting Fibrosis. Cells.

[43] Sassoli C., Vallone L., Tani A., Chellini F., Nosi D., Zecchi-Orlandini S. (2018). Combined use of bone marrow-derived mesenchymal stromal cells (BM-MSCs) and platelet rich plasma (PRP) stimulates proliferation and differentiation of myoblasts in vitro: New therapeutic perspectives for skeletal muscle repair/regeneration. Cell Tissue Res..

[44] Bernacchioni C., Ghini V., Squecco R., Idrizaj E., Garella R., Puliti E., Cencetti F., Bruni P., Donati C. (2021). Role of Sphingosine 1-Phosphate Signalling Axis in Muscle Atrophy Induced by TNFα in C2C12 Myotubes. Int. J. Mol. Sci..

[45] Formigli L., Sassoli C., Squecco R., Bini F., Martinesi M., Chellini F., Luciani G., Sbrana F., Zecchi-Orlandini S., Francini F. (2009). Regulation of transient receptor potential canonical channel 1 (TRPC1) by sphingosine 1-phosphate in C2C12 myoblasts and its relevance for a role of mechanotransduction in skeletal muscle differentiation. J. Cell Sci..

[46] Martella D., Mannelli M., Squecco R., Garella R., Idrizaj E., Antonioli D., Laus M., Wiersma D.S., Gamberi T., Paoli P. (2021). Cell instructive Liquid Crystalline Networks for myotube formation. iScience.

[47] Sassoli C., Pini A., Chellini F., Mazzanti B., Nistri S., Nosi D., Saccardi R., Quercioli F., Zecchi-Orlandini S., Formigli L. (2012). Bone marrow mesenchymal stromal cells stimulate skeletal myoblast proliferation through the paracrine release of VEGF. PLoS ONE.

[48] Luo D., Renault V.M., Rando T.A. (2005). The regulation of Notch signaling in muscle stem cell activation and postnatal myogenesis. Semin. Cell Dev. Biol..

[49] Formigli L., Meacci E., Sassoli C., Squecco R., Nosi D., Chellini F., Naro F., Francini F., Zecchi-Orlandini S. (2007). Cytoskeleton/stretch-activated ion channel interaction regulates myogenic differentiation of skeletal myoblasts. J. Cell. Physiol..

[50] Sin J., Andres A.M., Taylor D.J., Weston T., Hiraumi Y., Stotland A., Kim B.J., Huang C., Doran K.S., Gottlieb R.A. (2016). Mitophagy is required for mitochondrial biogenesis and myogenic differentiation of C2C12 myoblasts. Autophagy.

[51] Wagatsuma A., Sakuma K. (2013). Mitochondria as a potential regulator of myogenesis. Sci. World J..

[52] Semenza G.L. (2009). Regulation of oxygen homeostasis by hypoxia-inducible factor 1. Physiology.

[53] Summermatter S., Santos G., Pérez-Schindler J., Handschin C. (2013). Skeletal muscle PGC-1α controls whole-body lactate homeostasis through estrogen-related receptor α-dependent activation of LDH B and repression of LDH A. Proc. Natl. Acad. Sci. USA.

[54] Hood D.A., Memme J.M., Oliveira A.N., Triolo M. (2019). Maintenance of Skeletal Muscle Mitochondria in Health, Exercise, and Aging. Annu. Rev. Physiol..

[55] Ling M., Quan L., Lai X., Lang L., Li F., Yang X., Fu Y., Feng S., Yi X., Zhu C. (2021). VEGFB Promotes Myoblasts Proliferation and Differentiation through VEGFR1-PI3K/Akt Signaling Pathway. Int. J. Mol. Sci..

[56] Arsic N., Zacchigna S., Zentilin L., Ramirez-Correa G., Pattarini L., Salvi A., Sinagra G., Giacca M. (2004). Vascular endothelial growth factor stimulates skeletal muscle regeneration in vivo. Mol. Ther..

[57] Germani A., Di Carlo A., Mangoni A., Straino S., Giacinti C., Turrini P., Biglioli P., Capogrossi M.C. (2003). Vascular endothelial growth factor modulates skeletal myoblast function. Am. J. Pathol..

[58] Basic V.T., Jacobsen A., Sirsjö A., Abdel-Halim S.M. (2014). TNF stimulation induces VHL overexpression and impairs angiogenic potential in skeletal muscle myocytes. Int. J. Mol. Med..

[59] Rhoads R.P., Johnson R.M., Rathbone C.R., Liu X., Temm-Grove C., Sheehan S.M., Hoying J.B., Allen R.E. (2009). Satellite cell-mediated angiogenesis in vitro coincides with a functional hypoxia-inducible factor pathway. Am. J. Physiol. Cell Physiol..

[60] Hirota K., Semenza G.L. (2006). Regulation of angiogenesis by hypoxia-inducible factor 1. Crit. Rev. Oncol. Hematol..

[61] Olson N., van der Vliet A. (2011). Interactions between nitric oxide and hypoxia-inducible factor signaling pathways in inflammatory disease. Nitric Oxide.

[62] Déry M.A., Michaud M.D., Richard D.E. (2005). Hypoxia-inducible factor 1: Regulation by hypoxic and non-hypoxic activators. Int. J. Biochem. Cell Biol..

[63] Sibisi N.C., Snyman C., Myburgh K.H., Niesler C.U. (2022). Evaluating the role of nitric oxide in myogenesis in vitro. Biochimie.

[64] O’Hagan K.A., Cocchiglia S., Zhdanov A.V., Tambuwala M.M., Cummins E.P., Monfared M., Agbor T.A., Garvey J.F., Papkovsky D.B., Taylor C.T. (2009). PGC-1alpha is coupled to HIF-1alpha-dependent gene expression by increasing mitochondrial oxygen consumption in skeletal muscle cells. Proc. Natl. Acad. Sci. USA.

[65] Villa J.C., Chiu D., Brandes A.H., Escorcia F.E., Villa C.H., Maguire W.F., Hu C.J., de Stanchina E., Simon M.C., Sisodia S.S. (2014). Nontranscriptional role of Hif-1 α in activation of γ-secretase and notch signaling in breast cancer. Cell Rep..

[66] Sakagami H., Makino Y., Mizumoto K., Isoe T., Takeda Y., Watanabe J., Fujita Y., Takiyama Y., Abiko A., Haneda M. (2014). Loss of HIF-1α impairs GLUT4 translocation and glucose uptake by the skeletal muscle cells. Am. J. Physiol. Endocrinol. Metab..

[67] Hubbi M.E., Kshitiz G.D.M., Rey S., Wong C.C., Luo W., Kim D.H., Dang C.V., Levchenko A., Semenza G.L. (2013). A nontranscriptional role for HIF-1α as a direct inhibitor of DNA replication. Sci. Signal..

[68] Schmidt M., Schüler S.C., Hüttner S.S., von Eyss B., von Maltzahn J. (2019). Adult stem cells at work: Regenerating skeletal muscle. Cell. Mol. Life Sci..

[69] Yun Z., Lin Q., Giaccia A.J. (2005). Adaptive myogenesis under hypoxia. Mol. Cell. Biol..

[70] Zimowska M., Olszynski K.H., Swierczynska M., Streminska W., Ciemerych M.A. (2012). Decrease of MMP-9 activity improves soleus muscle regeneration. Tissue Eng. Part A.

[71] Kann A.P., Hung M., Wang W., Nguyen J., Gilbert P.M., Wu Z., Krauss R.S. (2022). An injury-responsive Rac-to-Rho GTPase switch drives activation of muscle stem cells through rapid cytoskeletal remodeling. Cell Stem Cell.

[72] Liu Z.Z.G., Taiyab A., West-Mays J.A. (2021). MMP9 Differentially Regulates Proteins Involved in Actin Polymerization and Cell Migration during TGF-β-Induced EMT in the Lens. Int. J. Mol. Sci..

[73] Bernacchioni C., Squecco R., Gamberi T., Ghini V., Schumacher F., Mannelli M., Garella R., Idrizaj E., Cencetti F., Puliti E. (2022). S1P Signalling Axis Is Necessary for Adiponectin-Directed Regulation of Electrophysiological Properties and Oxidative Metabolism in C2C12 Myotubes. Cells.

[74] Muratore M., Srsen V., Waterfall M., Downes A., Pethig R. (2012). Biomarker-free dielectrophoretic sorting of differentiating myoblast multipotent progenitor cells and their membrane analysis by Raman spectroscopy. Biomicrofluidics.

[75] Meacci E., Bini F., Sassoli C., Martinesi M., Squecco R., Chellini F., Zecchi-Orlandini S., Francini F., Formigli L. (2010). Functional interaction between TRPC1 channel and connexin-43 protein: A novel pathway underlying S1P action on skeletal myogenesis. Cell. Mol. Life Sci..

[76] Leikina E., Gamage D.G., Prasad V., Goykhberg J., Crowe M., Diao J., Kozlov M.M., Chernomordik L.V., Millay D.P. (2018). Myomaker and Myomerger Work Independently to Control Distinct Steps of Membrane Remodeling during Myoblast Fusion. Dev. Cell.

[77] Muller M., Schick M. (2011). An alternate path for fusion and its exploration by field-theoretic means. Curr. Top. Membr..

[78] Chernomordik L.V., Kozlov M.M. (2003). Protein-lipid interplay in fusion and fission of biological membranes. Annu. Rev. Biochem..

[79] Shimahara T., Bournaud R. (1991). Barium currents in developing skeletal muscle cells of normal and mutant mice foetuses with ‘muscular dysgenesis’. Cell Calcium.

[80] Tamayo T., Grajales L., García J. (2012). Commitment of satellite cells expressing the calcium channel α2δ1 subunit to the muscle lineage. J. Signal Transduct..

[81] Grajales L., Lach L.E., Janisch P., Geenen D.L., García J. (2015). Temporal Expression of Calcium Channel Subunits in Satellite Cells and Bone Marrow Mesenchymal Cells. Stem Cell Rev. Rep..

[82] García K., Nabhani T., García J. (2008). The calcium channel α2/δ1 subunit is involved in extracellular signaling. J. Physiol..

[83] Wang J., Weigand L., Lu W., Sylvester J.T., Semenza G.L., Shimoda L.A. (2006). Hypoxia Inducible Factor 1 Mediates Hypoxia-Induced TRPC Expression and Elevated Intracellular Ca^2+^ in Pulmonary Arterial Smooth Muscle Cells. Circ. Res..

[84] Yamazaki T., Mimura I., Kurata Y., Tanaka T., Nangaku M. (2023). Dznep, a histone modification inhibitor, inhibits HIF1α binding to TIMP2 gene and suppresses TIMP2 expression under hypoxia. Physiol. Rep..

[85] Chai M., Gu C., Shen Q., Liu J., Zhou Y., Jin Z., Xiong W., Zhou Y., Tan W. (2020). Hypoxia alleviates dexamethasone-induced inhibition of angiogenesis in cocultures of HUVECs and rBMSCs via HIF-1α. Stem Cell Res. Ther..

[86] Hu J., Wang W., Zhang F., Li P.L., Boini K.M., Yi F., Li N. (2017). Hypoxia inducible factor-1α mediates the profibrotic effect of albumin in renal tubular cells. Sci. Rep..

[87] Takahara Y., Tokunou T., Kojima H., Hirooka Y., Ichiki T. (2017). Deletion of hypoxia-inducible factor-1α in myeloid lineage exaggerates angiotensin II-induced formation of abdominal aortic aneurysm. Clin. Sci..

[88] Kai A.K., Chan L.K., Lo R.C., Lee J.M., Wong C.C., Wong J.C., Ng I.O. (2016). Down-regulation of TIMP2 by HIF-1α/miR-210/HIF-3α regulatory feedback circuit enhances cancer metastasis in hepatocellular carcinoma. Hepatology.

[89] Schelter F., Halbgewachs B., Bäumler P., Neu C., Görlach A., Schrötzlmair F., Krüger A. (2011). Tissue inhibitor of metalloproteinases-1-induced scattered liver metastasis is mediated by hypoxia-inducible factor-1α. Clin. Exp. Metastasis.

[90] Leeman M.F., Curran S., Murray G.I. (2002). The structure, regulation, and function of human matrix metalloproteinase-13. Crit. Rev. Biochem. Mol. Biol..

[91] Lei H., Leong D., Smith L.R., Barton E.R. (2013). Matrix metalloproteinase 13 is a new contributor to skeletal muscle regeneration and critical for myoblast migration. Am. J. Physiol. Cell Physiol..

