# Supplementary figures and images for: HIF-1α/MMP-9 Axis Is Required in the Early Phases of Skeletal Myoblast Differentiation under Normoxia Condition In Vitro
Source: Cells. 2023 Dec 16;12(24):2851. doi: 10.3390/cells12242851 (PMC10742321; doi:10.3390/cells12242851)

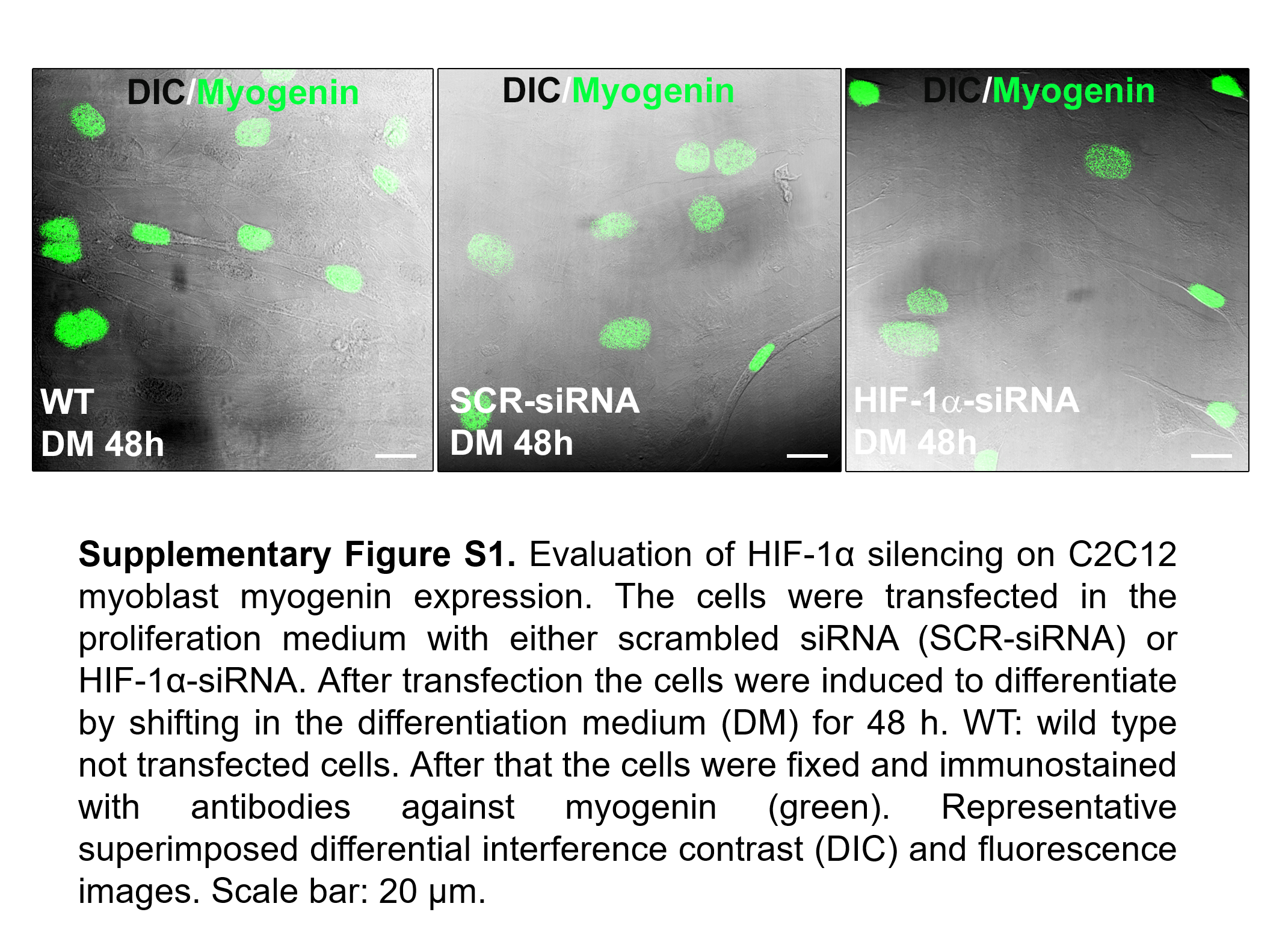

Supplement: Supplementary file 1 [file cells-12-02851-s001.zip › cells-2731385-supplementary.tif]
